# Molecular Precision Medicine: Application of Physiologically Based Pharmacokinetic Modeling to Predict Drug–Drug Interactions Between Lidocaine and Rocuronium/Propofol/Paracetamol

**DOI:** 10.3390/ijms26041506

**Published:** 2025-02-11

**Authors:** Abigail Silva, Joana Mourão, Nuno Vale

**Affiliations:** 1 PerMed Research Group, RISE-Health, Faculty of Medicine, University of Porto, Alameda Professor Hernâni Monteiro, 4200-319 Porto, Portugal; abigailsilva@outlook.pt; 2 Laboratory of Personalized Medicine, Department of Community Medicine, Health Information and Decision (MEDCIDS), Faculty of Medicine, University of Porto, Rua Doutor Plácido da Costa, 4200-450 Porto, Portugal; 3 Department of Anesthesiology, Centro Hospitalar Universitário de São João, Alameda Professor Hernâni Monteiro, 4200-319 Porto, Portugal; joanamourao@med.up.pt; 4 RISE-Health, Surgery and Physiology Department, Faculty of Medicine, University of Porto, Rua Doutor Plácido da Costa, 4200-450 Porto, Portugal; 5 RISE-Health, Department of Community Medicine, Health Information and Decision (MEDCIDS), Faculty of Medicine, University of Porto, Rua Doutor Plácido da Costa, 4200-450 Porto, Portugal

**Keywords:** lidocaine, anesthetics, Hep G2 cells, PBPK model, drug–drug interactions

## Abstract

The perioperative period, encompassing preoperative, intraoperative, and postoperative phases, is crucial for comprehensive patient care. During this time, the use of opioids and other drugs can lead to drug–drug interactions (DDIs), potentially resulting in adverse drug reactions (ADRs) that increase morbidity, mortality, and healthcare costs. This study investigates the drug–drug interactions (DDIs) between rocuronium, propofol, paracetamol, and lidocaine, focusing on the CYP-mediated metabolism of these drugs in the perioperative context, where these drugs are frequently co-administered. Using physiologically based pharmacokinetic (PBPK) modeling through the GastroPlus™ software and in vitro experiments with Hep G2 cells, we aimed to assess potential toxicities and pharmacokinetic interactions. Cellular viability assays revealed significant toxicity when lidocaine was combined with propofol and rocuronium, while paracetamol exhibited no considerable impact on viability. PBPK simulations confirmed moderate interactions with rocuronium and weak interactions with propofol but no relevant interactions with paracetamol. These findings emphasize the need for dose adjustments in perioperative settings to enhance patient safety, particularly with propofol and rocuronium, while supporting the co-administration of lidocaine and paracetamol. These findings show the importance of moving towards a personalized medicine model, adjusting the clinical use of lidocaine according to individual patient needs, thus promoting safer and more effective perioperative care and moving beyond the “one-size-fits-all” approach in anesthetic management.

## 1. Introduction

The perioperative period includes the preoperative, intraoperative, and postoperative phases and is a critical period when patients are prepared for surgery, receive anesthesia, and are monitored during recovery. This period is crucial for providing comprehensive care to patients, from physical and emotional preparation to post-surgical recovery [1]. During this phase, the use of drugs is common to control pain, sedate the patient, and prevent infections, however, these drugs can lead to drug–drug interactions (DDIs) that cause adverse drug reactions (ADRs), potentially increasing morbidity, mortality, and healthcare costs. To minimize these DDIs, dose adjustments, changes in drug selection, or additional monitoring to ensure patient safety can be the key [2,3,4,5,6].

Drug interactions in the perioperative context can occur through several mechanisms, such as distribution, metabolism, or excretion, which are critical factors for the success of anesthetic therapies and can impact both the efficacy and safety of treatments [7,8]. Obtaining a complete medical history, including a list of all drugs the patient is taking, is essential for predicting and preventing potential drug interactions. Also, the use of physiologically based pharmacokinetic (PBPK) models can help predict drug interactions and guide dose adjustments in clinical practice [9].

**Figure 1:**
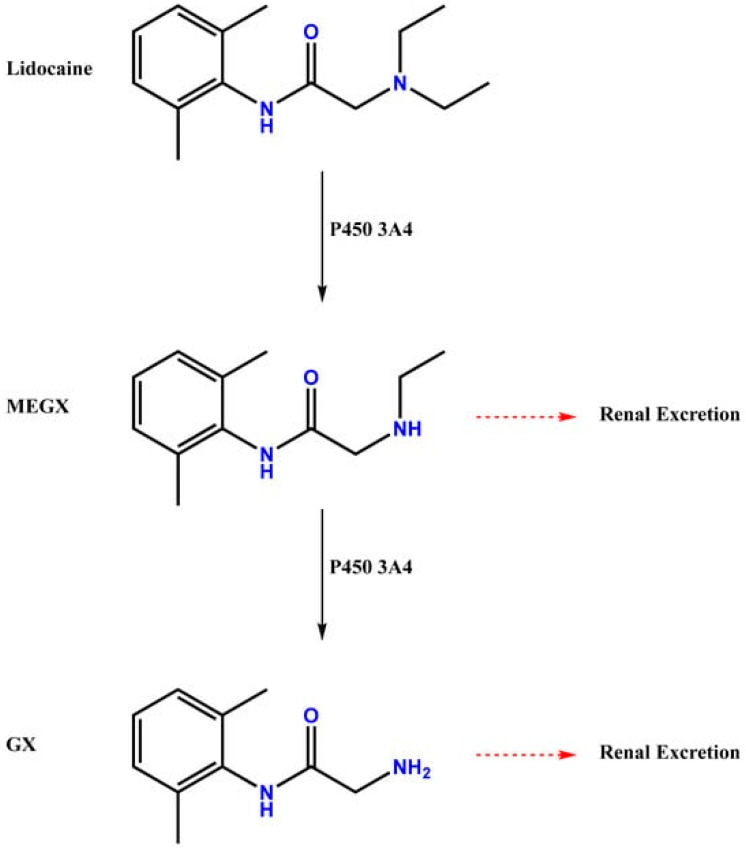
Chemical structure of lidocaine and metabolism pathway.

In this study, lidocaine, propofol, paracetamol, and rocuronium are widely used drugs in surgical procedures [10,11,12,13]. Propofol is a common intravenous anesthetic used to induce and maintain general anesthesia [11]. It acts mainly on the central nervous system, facilitating the action of the inhibitory neurotransmitter gamma-aminobutyric acid (GABA) on GABA-A receptors [14]. This mechanism promotes a sedative and anesthetic effect. It is rapidly distributed throughout the tissues and metabolized in the liver and has a high lipophilicity, which allows it to pass quickly through the blood–brain barrier. Its elimination half-life is relatively short, and this facilitates rapid recovery after cessation of administration. It is mainly metabolized by *CYP2B6* and *CYP3A4* [11,15,16,17]. Rocuronium is a non-depolarizing neuromuscular blocker that provides muscle relaxation. It acts on the neuromuscular system by antagonizing the acetylcholine receptors at the neuromuscular junction, preventing the transmission of the nerve impulse and resulting in muscle paralysis [12]. It is well absorbed and distributed throughout the tissues after intravenous administration. It is mainly metabolized by the liver and excreted in the bile. Its half-life of action is intermediate, with effects lasting from 30 to 60 min, depending on the dose administered. Interactions with other drugs that inhibit its metabolism, such as lidocaine, can increase its concentration and neuromuscular effect [18,19,20,21,22]. Paracetamol is an analgesic and antipyretic used to relieve pain and reduce fever. It acts mainly on the central nervous system, where it inhibits the synthesis of prostaglandins in the brain, helping to reduce pain and fever. Unlike other anti-inflammatory drugs, paracetamol has little peripheral anti-inflammatory activity [13,23,24]. It is rapidly absorbed from the gastrointestinal tract and widely distributed throughout body tissues. It is metabolized mainly in the liver and excreted in the urine. The half-life of paracetamol is generally between 1 and 4 h, depending on the dose and the liver condition. Excessive doses or prolonged use can cause hepatoxicity, especially in individuals with impaired liver function. Concomitant use with other drugs that induce *CYP2B6* can increase the clearance of paracetamol, reducing its effectiveness [24,25,26,27]. Lidocaine stands as the central drug in this study as it is consistently employed and combined with the other three drugs. This drug is an amide anesthetic that is used locally. Initially, it was used intravenously as an antiarrhythmic drug, but later, it was suggested that intravenous (IV) lidocaine exhibited analgesic properties that would be advantageous in perioperative situations and in reducing the pain of propofol administration. Since then, lidocaine has been extensively utilized in several medical practices as an antiarrhythmic and local anesthetic [10,28,29,30]. It is widely used in healthcare because of its proven ability to reduce pain and prevent cardiac arrhythmias. Based on the Vaughan–Williams classification system, lidocaine falls within the class Ib of antiarrhythmic agents. Its chemical structure is defined by an amine group and an aromatic ring connected to an aliphatic chain; its molecular formula is C_14_H_22_N_2_O (Figure 1). The monocarboxylic acid amide, known as lidocaine, is produced when N, N-diethylglycine, and 2,6-dimethylaniline are formally condensed. By directly affecting peripheral nerves, it relieves localized or regional pain and is frequently utilized during the perioperative phase [31,32,33,34].

A literature review of the CYPs of the drugs to be tested in combination with lidocaine was carried out, and it showed that lidocaine, propofol, rocuronium, and paracetamol share common metabolic pathways and are all metabolized by enzymes of the cytochrome P450 system (CYP) (Table 1). The fact that lidocaine, paracetamol, propofol, and rocuronium are metabolized by cytochrome P450 enzymes can lead to significant interactions between these drugs. Therefore, the common metabolization of these drugs by CYPs can have important repercussions and lead to interactions that affect the safety and efficacy of treatment [35,36,37,38].

Although lidocaine was introduced to hospitals as an effective and safe local anesthetic for a variety of medical procedures, and the initial studies of this drug concluded that it was a safe drug for short procedures, its use has been questioned ever since [45,46]. Given the frequent use of these drugs in clinical practice at ULS São João and the lack of comprehensive studies addressing their interactions, it is important to investigate these potential DDIs to enhance patient safety and treatment efficacy. This study aims to apply physiologically based pharmacokinetic (PBPK) modeling to predict DDIs between lidocaine, propofol, rocuronium, and paracetamol, focusing on the CYP-mediated metabolism of these drugs in the perioperative period. This approach aims to improve the safety and efficacy of perioperative care, moving towards a model of personalized medicine. Personalized medicine aims to optimize treatment based on each patient’s unique characteristics. Lidocaine can play a crucial role in achieving this by enabling more precise control of anesthesia based on factors such as pain sensitivity, metabolism, and the patient’s medical history. Instead of administering a standardized dose, physicians can adjust the concentration and quantity of the drugs based on the patient’s response, resulting in more effective and comfortable anesthesia [47,48]. So, to investigate the efficacy and safety of the combinations of lidocaine with propofol, rocuronium, and paracetamol, an integrated approach combining computational modeling and in vitro experimentation was adopted. The PBPKPlus™ module in the GastroPlus™ software was used to model the pharmacokinetics (PKs) of lidocaine and the other three drugs [49], while in vitro experiments were conducted with Hep G2 cells, a human hepatocellular carcinoma cell line widely used as a model for studying human hepatic metabolism [50].

GastroPlus™ is a simulation software that allows for modeling the absorption, distribution, metabolism, and excretion (ADME) of drugs in the human body. It can simulate how different drugs interact with each other and predict how these interactions may affect their pharmacokinetic profiles and, consequently, their efficacy and safety. Through simulations, it is possible to identify changes in drug plasma concentrations due to inhibition or induction of cytochrome P450 (CYP) enzymes involved in their metabolism, helping predict potential adverse events and adjust doses to optimize combination therapy. These studies are a crucial tool in predicting these interactions and guiding dose adjustments in clinical practice. The predictive performance of PBPKs for CYP-mediated drug interactions has already been widely established, and many PBPK simulations have been accepted by regulatory agencies as the basis for dose adjustments in drug labeling [51,52,53,54].

Complementing these simulations, in vitro experiments with the Hep G2 cells focussed on the metabolic interactions between lidocaine, propofol, rocuronium, and paracetamol. Hep G2 cells are a well-established model for studying drug metabolism and liver toxicity due to their expression of CYP enzymes [55]. By exposing these cells to the drug combinations, we aimed to observe the metabolic pathways and identify any significant interactions that could affect drug efficacy and safety. Hep G2 cells express several cytochrome P450 enzymes. These in vitro models were exposed to combinations of lidocaine, propofol, rocuronium, and paracetamol to identify significant interactions that could affect the efficacy and safety of the drugs [55,56]. This study leveraged the PBPKPlus™ module in GastroPlus™ to simulate lidocaine’s pharmacokinetics (PKs) and its interactions with propofol, rocuronium, and paracetamol. Additionally, in vitro experiments using Hep G2 cells, a model for human liver metabolism, were conducted to validate these simulations and provide insights into the metabolic interactions at the cellular level. We are conducting this study because a limited body of significant studies demonstrate the safety of concomitant use of these drugs, despite their common usage in clinical practice. This study aims to investigate the impact of three drugs, rocuronium, propofol, and paracetamol, on lidocaine exposure and to evaluate the efficacy of the combinations of lidocaine/propofol, lidocaine/paracetamol, and lidocaine/rocuronium. The focus is on understanding how rocuronium, propofol, and paracetamol affect the CYP-mediated metabolism of lidocaine.

## 2. Results

### 2.1. Experimental Results: In Vitro Hep G2 Cells

In this study, we intend to investigate the combination of propofol, rocuronium, and paracetamol when administered with lidocaine to evaluate their interactions and possible DDIs. To carry out this study, the Hep G2 cell line was chosen for use. This is a human hepatocellular carcinoma cell line widely used in scientific studies [55].

The objective is to demonstrate that the combination of the drugs mentioned is safe. Cell viability will be evaluated using the MTT assay, and, additionally, to supplement the research with a more thorough examination, LC-MS will be performed to examine the supernatant of the samples whose combinations have the highest concentrations. To have a more complete and real model, each drug was tested alone in the cells for 24, 48, and 72 h at four different concentrations (50, 100, 250, and 500 µM), then each of the drugs, rocuronium, propofol, and paracetamol, were tested in combination with lidocaine, also for 24, 48 and 72 h, at the two strongest concentrations (250 and 500 µM). The half maximal inhibitory concentration (IC50) of the drugs tested was not calculated, as these compounds are already used in clinical practice with well-established doses and therapeutic regimens, and for this reason, the calculation of IC50 would not yield meaningful or relevant values, as the doses employed are predefined and do not correspond to variable concentrations typically required to determine an IC50. Instead, the research evaluated clinically significant parameters, such as the drug combinations’ efficacy, pharmacokinetic stability, and potential interactions between the compounds.

#### 2.1.1. The Effects of Lidocaine, Propofol, Paracetamol, and Rocuronium on Hep G2 Cells After 24 h

The primary focus was to determine whether these drugs induced significant changes in cell viability and morphology.

**Figure 2:**
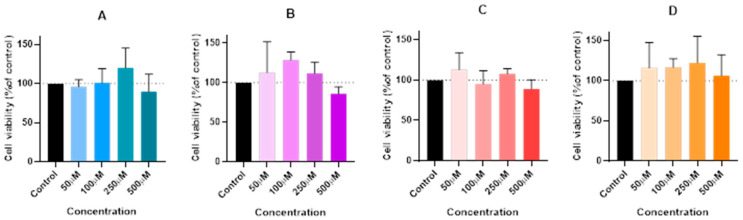
Effect of lidocaine (**A**), propofol (**B**), paracetamol (**C**), and rocuronium (**D**) on the viability of the Hep G2 cells 24 h after the drug was added to the cells. Each drug was added in a fresh medium. The results are presented as the mean ± SD, representing the cell viability (% of control) of the 3 experiments (n = 3). Although Graphs (**A**–**D**) indicate no significant differences in the results, suggesting a minimal impact on cell viability, it is noteworthy that there is a slight decrease in cell viability observed at the higher concentrations of all the drugs.

**Figure 3:**
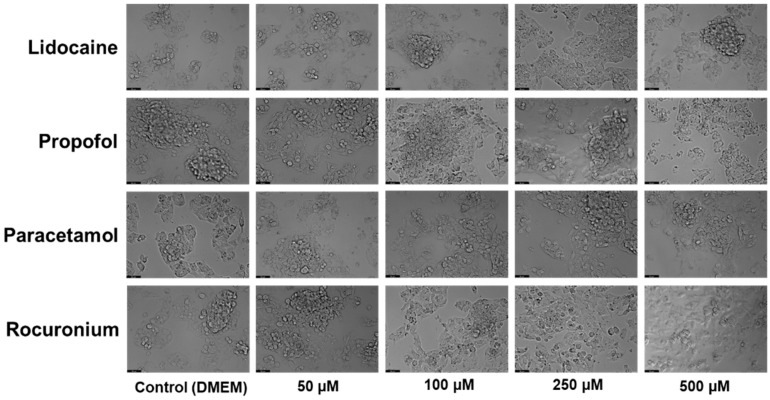
Effect of lidocaine, propofol, paracetamol, and rocuronium on the morphology of the Hep G2 cells 24 h after the drug was added to the cells. The cells were treated with 50, 100, 250, and 500 μM of each drug and DMEM (control). Scale bar: 50 μm.

For that, the Hep G2 cells were cultured and treated with 50, 100, 250, and 500 µM concentrations of lidocaine (A), propofol (B), paracetamol (C), and rocuronium (D) alone. The exposure duration was standardized to 24 h for all treatments. Cell viability was assessed post-treatment using an MTT assay (Figure 2), and cellular morphology was observed under a microscope (Figure 3).

Proceeding with the analysis of the results, it is clear that the graphical data on cell viability showed no significant differences between the treated groups and the control. Statistical analysis confirmed that the changes in cell viability following exposure to propofol, lidocaine, rocuronium, and paracetamol were not statistically significant. This suggests that, within the tested concentrations, these drugs do not negatively affect the viability of the Hep G2 cells. Microscopic images were taken to assess any potential cell morphological changes after treatment. Consistent with the viability results, the images did not reveal noticeable changes in the structure or morphology of the treated cells compared to the control. The cellular architecture remained stable and uniform, indicating that these drugs did not induce any morphological damage or stress. This suggests that in short-term exposure, these drugs pose minimal risk of hepatic toxicity, reinforcing their safety when used together in perioperative settings, at least under the in vitro conditions tested.

#### 2.1.2. The Effects of Lidocaine, Propofol, Paracetamol, and Rocuronium on Hep G2 Cells After 48 h

**Figure 4:**
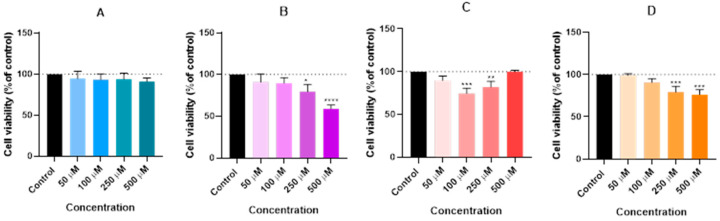
Effect of lidocaine (**A**), propofol (**B**), paracetamol (**C**), and rocuronium (**D**) on the viability of the Hep G2 cells 48 h after the drugs were added to the cells. Each drug was added in fresh medium. The results are presented as the mean ± SD, representing the cell viability (% of control) of the 3 experiments (n = 3). **** *p* < 0.0001, *** *p* < 0.001, ** *p* < 0.01, and * *p* < 0.05 vs. the control. Graph (**A**) shows no significant differences, but Graphs (**B**–**D**) show otherwise. Graph (**B**) displays a very significant reduction in cell viability at higher doses. Graph (**C**) shows minimal impact on cell viability, affirming its safety profile with higher concentrations, and Graph (**D**) reflects a more significant decline in cell viability compared to the 24-h mark, particularly at higher doses.

**Figure 5:**
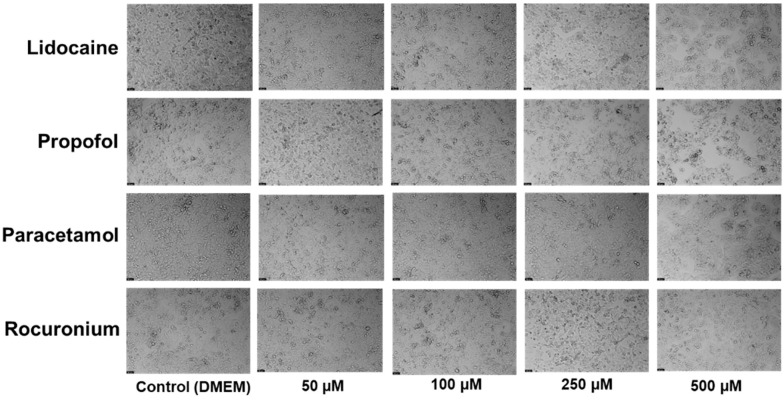
Effect of lidocaine, propofol, paracetamol, and rocuronium on the morphology of the Hep G2 cells, 48 h after the drug was added to the cells. The cells were treated with 50, 100, 250, and 500 μM of each drug and DMEM (control). Scale bar: 50 μm.

To further the investigation, the same process was used to track the impact after 48 h. The goal was to assess the effects of the four drugs (propofol, lidocaine, rocuronium, and paracetamol) on the Hep G2 cells after 48 h, similar to the 24-h experience. The Hep G2 cells were cultivated and exposed to the same concentrations used in the 24-h experiment (50, 100, 250, and 500 µM) of lidocaine (A), propofol (B), paracetamol (C), and rocuronium (D). Following treatment, an MTT assay was used to determine cell viability (Figure 4), and microscopy was used to look at cellular morphology (Figure 5).

The results showed that propofol, at concentrations of 250 µM and 500 µM, led to significant reductions in cell viability, with statistical significance levels of *p* < 0.05 and *p* < 0.0001, respectively. Paracetamol also demonstrated substantial effects at 100 µM and 250 µM, with *p*-values indicating *p* < 0.01 and *p* < 0.001. Similarly, rocuronium, at its higher concentrations of 250 µM and 500 µM, showed a strong significance level (*p* < 0.001). Despite these viability changes, the microscopic images revealed that the overall morphology of the Hep G2 cells remained unaffected across all treatment groups. However, there was a slight decrease in cell density observed in the wells where significant changes in viability were recorded. This indicates that while the cellular structure itself was preserved, the number of viable cells diminished in response to higher doses of propofol, paracetamol, and rocuronium.

In contrast, lidocaine did not significantly affect cell viability over the 48-h exposure period, even at higher concentrations. Propofol, paracetamol, and rocuronium, however, showed a marked reduction in cell viability, which points to their stronger cytotoxic potential at elevated doses. Notably, despite the reductions in viability, the integrity of the cell morphology remained intact across all treatment groups, suggesting that the cytotoxic effects were more selective, affecting cell survival without causing widespread structural damage.

#### 2.1.3. The Effects of Lidocaine, Propofol, Paracetamol, and Rocuronium on Hep G2 Cells After 72 h

An MTT assay was made 72 h after the drugs were administered to conclude the research on the effects of lidocaine, propofol, paracetamol, and rocuronium alone on the Hep G2 cells. This experiment was designed to evaluate the effects of these three drugs on the Hep G2 cells over 72 h, and the primary aim was to evaluate changes in cell viability and morphology over this extended exposure.

**Figure 6:**
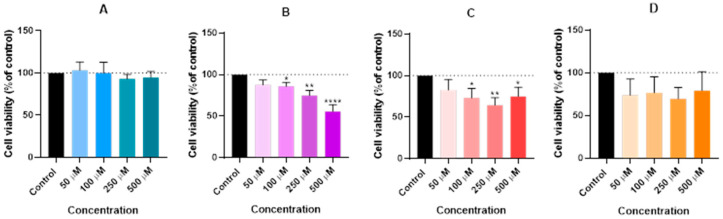
Effect of lidocaine (**A**), propofol (**B**), paracetamol (**C**), and rocuronium (**D**) on the viability of the Hep G2 cells 72 h after the drugs were added to the cells. Each drug was added in fresh medium. The results are presented as the mean ± SD, representing the cell viability (% of control) of the 3 experiments (n = 3). **** *p* < 0.0001, ** *p* < 0.01, * *p* < 0.05 vs. the control. Graph (**A**) shows a consistent trend of no significant impact on cell viability, remaining less cytotoxic compared to the other drugs, while Graph (**B**) continues to exhibit a significant decrease in cell viability. Graph (**C**) maintains its safety profile, showing no significant effects on cell viability even after extended exposure to higher concentrations, and Graph (**D**) reveals a marked decline in cell viability at higher concentrations, even though the analyses indicate no significant differences.

**Figure 7:**
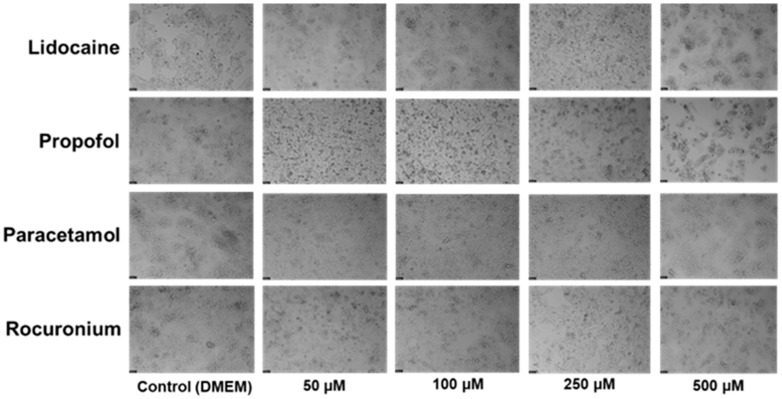
Effect of lidocaine, propofol, paracetamol, and rocuronium on the morphology of the Hep G2 cells 72 h after the drug was added to the cells. The cells were treated with 50, 100, 250, and 500 μM of each drug and DMEM (control). Scale bar: 50 μm.

The Hep G2 cells were cultured and treated with the same concentrations of propofol, lidocaine, rocuronium, and paracetamol used before (24 h and 48 h) for 72 h. After the treatment period, cell viability was measured using an MTT assay (Figure 6), and cell morphology was examined through microscopy (Figure 7).

The results showed that neither lidocaine nor rocuronium produced significant changes in cell viability, even at higher concentrations. However, propofol displayed a clear dose-dependent impact on viability, with statistically significant reductions at 100 µM, 250 µM, and 500 µM, showing *p* < 0.05, *p* < 0.01, and *p* < 0.0001, respectively. Paracetamol also exhibited significant effects on cell viability at these concentrations, though to a lesser extent, with significance levels of *p* < 0.05 at 100 µM and *p* < 0.01 at 250 µM and 500 µM. Despite these reductions in cell viability, microscopic observations confirmed that the overall morphology of the Hep G2 cells remained unchanged. However, a noticeable decrease in cell density was observed as drug concentrations increased, particularly in the wells treated with higher concentrations of propofol and paracetamol. This suggests that while the structure of the cells was unaffected, the number of viable cells was significantly reduced in response to these higher concentrations.

Over the 72-h exposure period, it became evident that lidocaine has minimal cytotoxic effects on the Hep G2 cells, reinforcing its safer profile in terms of cell viability. In contrast, propofol and paracetamol demonstrated significant reductions in cell viability at all tested concentrations. Despite these reductions, the morphological integrity of the cells was preserved, indicating that the cytotoxicity of propofol and paracetamol was selective, as it affected the number of cells rather than their structural health.

#### 2.1.4. The Effects of Lidocaine in Combination with Propofol on Hep G2 Cells After 24, 48, and 72 h

Following the previous experiments, new tests were conducted on cells with multiple drug combinations. In these new results, lidocaine is consistently used together with the remaining three drugs. The combination of lidocaine and propofol in these new results was observed at 24, 48, and 72 h after the drugs were added to the cells.

**Figure 8:**
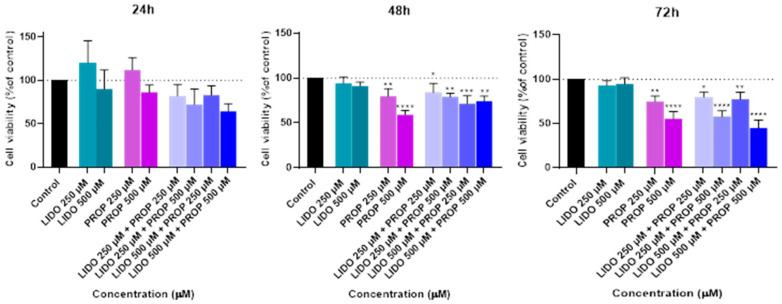
Effect of lidocaine in combination with propofol on the viability of the Hep G2 cells 24, 48, and 72 h after the drugs were added to the cells. Each drug was added in fresh medium. The results are presented as the mean ± SD and represent the viability of cells (% of control) of the 3 experiments (n = 3). **** *p* < 0.0001, *** *p* < 0.001, ** *p* < 0.01, and * *p* < 0.05 vs. the control.

**Figure 9:**
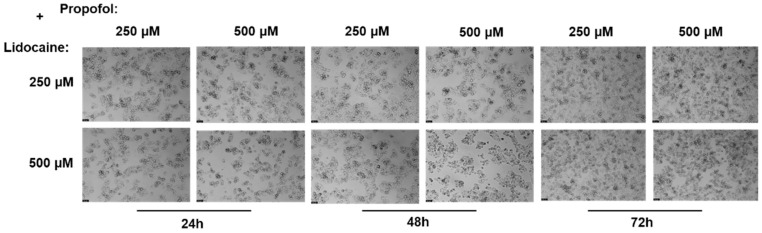
Effect of lidocaine combined with propofol on the viability of the Hep G2 cells 24, 48, and 72 h after the drug was added to the cells. Scale bar: 50 μm.

Following this treatment, the MTT assay was used to measure the viability of the cells (Figure 8). In addition, a microscope examination of the cell morphology allowed for a visual evaluation of the structural alterations induced by the combination of propofol and lidocaine treatment (Figure 9).

The MTT assay results show that, although there were no significant differences in cell viability at the 24-h mark when comparing the combination of propofol and lidocaine to the controls or to the drugs individually, things changed significantly over time. At 48 h, we began to see a clear reduction in cell viability in some of the combinations. Specifically, the combinations of lidocaine 500 µM with propofol 250 µM and lidocaine 500 µM with propofol 500 µM, which resulted in a significant decrease in viability compared to both the control and the drugs used individually. This points to a negative synergistic effect, where the combination of these two drugs appears to have a stronger cytotoxic impact after 48 h of exposure than when they are administered alone.

By 72 h, these effects became even more pronounced. All combinations of lidocaine and propofol, especially at higher concentrations, such as 500 µM of each, showed a marked reduction in cell viability, even more severe than at 48 h. This further reinforces the idea that there is a negative synergistic interaction between these drugs, which seems to intensify with longer exposure times. These findings are critical because they suggest that the combined use of lidocaine and propofol could significantly impact cell viability over time, which may have important implications in clinical settings, particularly during prolonged surgical procedures or extended drug administration.

Microscopic analysis provided additional insight into these effects. While no significant changes in cell morphology were observed—meaning that the overall shape and structure of the cells remained consistent across all treatment conditions—there was a noticeable decrease in cell density as drug concentrations and exposure time increased. This is particularly evident in the higher concentration combinations of lidocaine and propofol, where the decrease in cell density mirrors the significant drop in cell viability observed in the MTT assay. This suggests that, despite the cells maintaining their structure, their overall number is dramatically reduced, indicating that the combination of these drugs is leading to cell death or reduced proliferation over time, and the substantial decrease in cell density and viability highlights the importance of carefully monitoring the use of these drug combinations in clinical practice, especially during long-term treatments.

#### 2.1.5. The Effects of Lidocaine in Combination with Paracetamol on Hep G2 Cells After 24, 48, and 72 h

**Figure 10:**
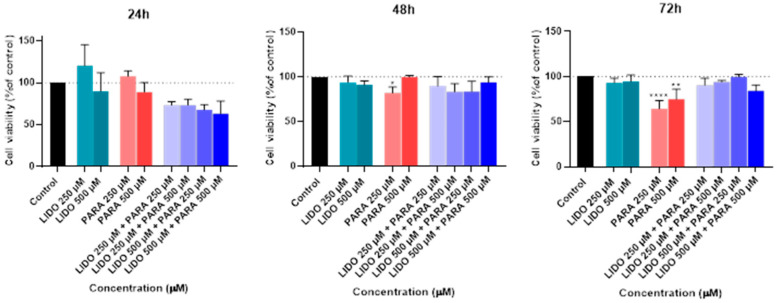
Effect of lidocaine in combination with paracetamol on the viability of the Hep G2 cells 24, 48, and 72 h after the drugs were added to the cells. Each drug was added in fresh medium. The results are presented as the mean ± SD, representing the cell viability (% of control) of the 3 experiments (n = 3). **** *p* < 0.0001, ** *p* < 0.01, and * *p* < 0.05 vs. the control.

**Figure 11:**
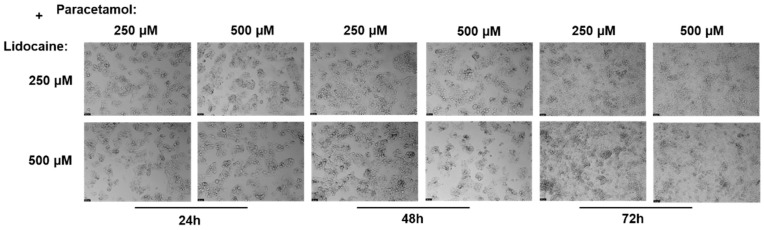
Effect of lidocaine combined with paracetamol on the viability of the Hep G2 cells 24, 48, and 72 h after the drug was added to the cells. Scale bar: 50 μm.

Continuing from the previous experiments, new tests were conducted to investigate the effects of lidocaine in combination with paracetamol on the Hep G2 cells over 24, 48, and 72 h. In these new experiments, lidocaine was tested in combination with paracetamol. Following this treatment, the MTT assay was employed to measure cell viability (Figure 10). Additionally, a microscopic examination of cell morphology was performed to visually assess any structural changes induced by the lidocaine and paracetamol combination (Figure 11).

The results from the MTT assay showed that the combination of lidocaine and paracetamol did not lead to any significant changes in cell viability at 24, 48, or 72 h when compared to the controls or the drugs administered individually. This indicates that, over time, the combination of these two drugs does not exhibit a pronounced synergistic or antagonistic effect on cell viability. Essentially, their combined use in the tested concentrations appears to have no major impact on the health or survival of the Hep G2 cells, reinforcing the idea that these drugs are safe to use together in terms of cytotoxicity.

Further support comes from the analysis of the microscope images, which revealed that there were no notable changes in cell morphology throughout the exposure periods. The shape and structure of the cells remained consistent across all conditions, and cell density also remained relatively stable, even at higher concentrations or longer exposure times. These observations are in line with the MTT data, indicating that the combination of lidocaine and paracetamol does not significantly affect cellular morphology or density, further suggesting that this drug pairing does not pose any additional risks to cell viability under the conditions tested.

#### 2.1.6. The Effects of Lidocaine in Combination with Rocuronium on Hep G2 Cells After 24, 48, and 72 h

Continuing from the previous experiments, the final combination tested was lidocaine in combination with rocuronium on the Hep G2 cells, evaluated at 24, 48, and 72 h.

**Figure 12:**
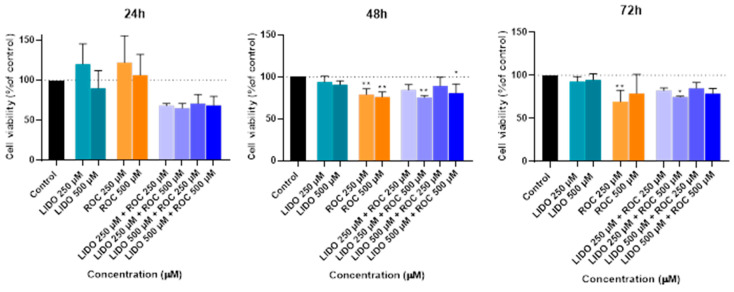
Effect of lidocaine in combination with rocuronium on the viability of the Hep G2 cells 24, 48, and 72 h after the drugs were added to the cells. Each drug was added in fresh medium. The results are presented as the mean ± SD, representing the cell viability (% of control) of the 3 experiments (n = 3). ** *p* < 0.01, and * *p* < 0.05 vs. the control.

**Figure 13:**
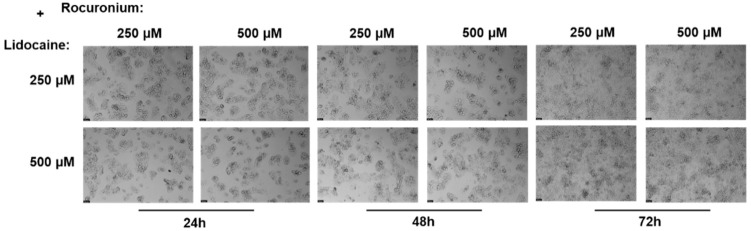
Effect of lidocaine combined with rocuronium on the viability of the Hep G2 cells 24, 48, and 72 h after the drug was added to the cells. Scale bar: 50 μm.

Following this treatment, the MTT assay was employed to measure cell viability (Figure 12). Additionally, a microscopic examination of cell morphology was performed to visually assess any structural changes induced by the lidocaine and rocuronium combination (Figure 13).

The MTT assay results indicated that at the 24-h mark, there were no significant differences in cell viability between the combinations of lidocaine and rocuronium compared to the controls or the individual drugs. However, at 48 h, a slight but noticeable difference in cell viability emerged, particularly with the combination of lidocaine at 250 µM and rocuronium at 500 µM, which showed a statistically significant reduction in viability compared to both the control and the individual drug treatments. By the 72-h time point, the results mirrored those at 24 h, with minimal significant changes in viability, except for the same combination (LIDO 250 µM + ROC 500 µM), which continued to show a slight but significant decrease in cell viability compared to the other conditions. Microscopic analysis reinforced these findings. As with the combination of lidocaine and paracetamol, there were no significant changes in cell morphology, and the overall structure of the cells remained intact across all treatment groups. Additionally, cell density showed minimal changes, suggesting that the combination of lidocaine and rocuronium does not drastically affect cell proliferation or cause major cellular stress. However, the slight reduction in viability observed with the LIDO 250 µM + ROC 500 µM combination suggests that certain concentrations of these drugs, when used together, might have a mild negative impact on cell health over time. Although the combination of lidocaine and rocuronium exhibits a generally safe profile, with minimal impact on cell morphology and density, the slight reductions in cell viability observed at specific concentrations over longer exposure periods indicate that this combination may require careful dosing to avoid potential cytotoxicity, particularly in more prolonged treatments or higher doses.

### 2.2. LC-MS Analyses

**Figure 14:**
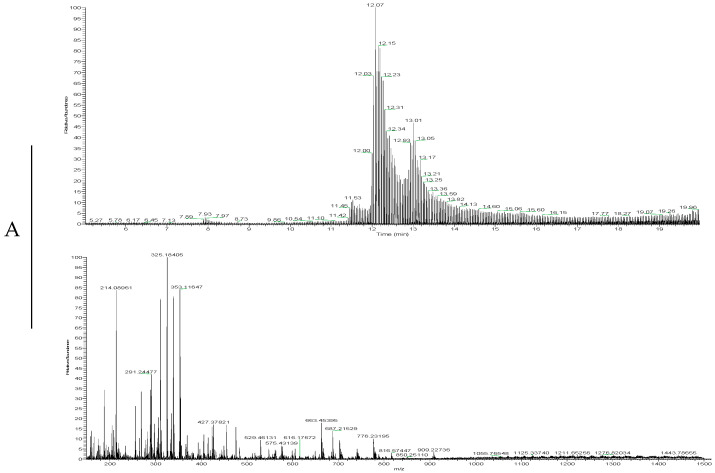

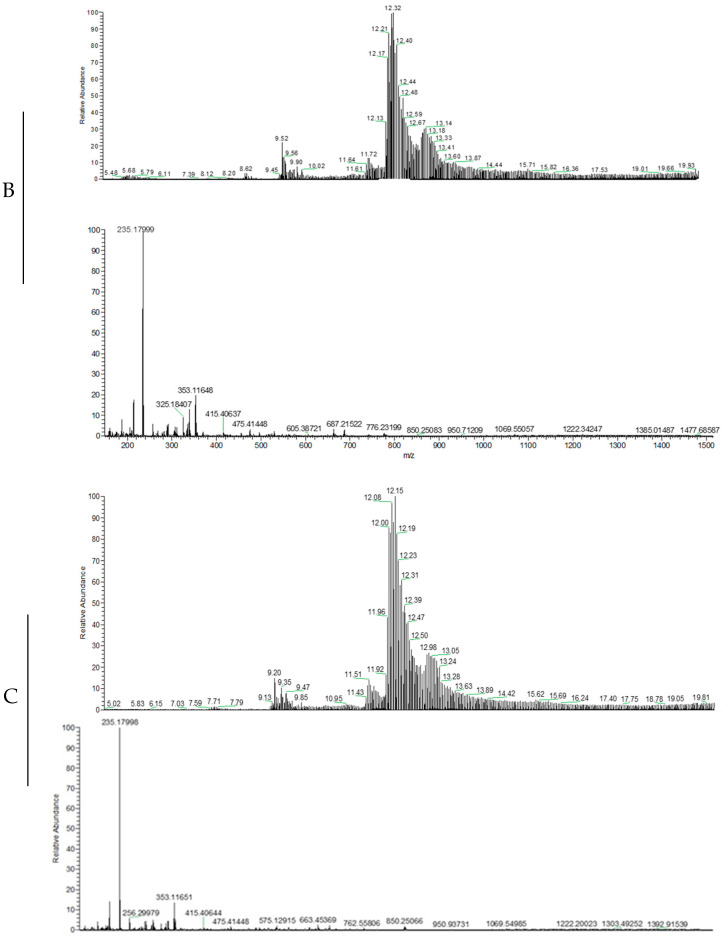

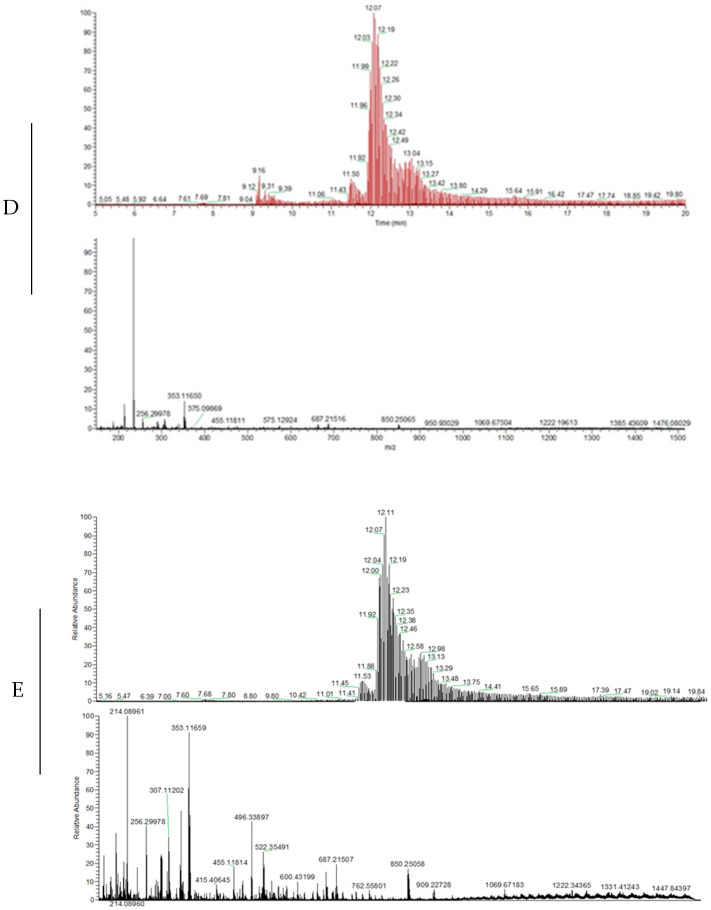

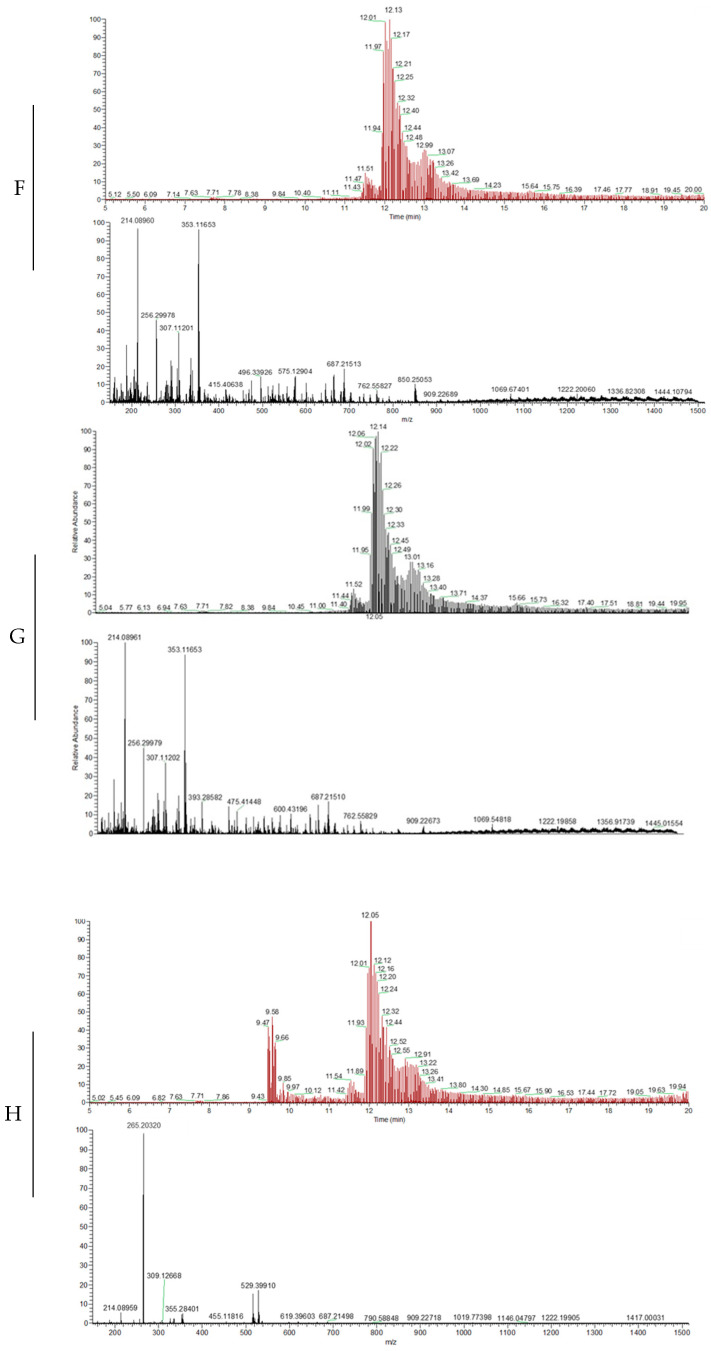

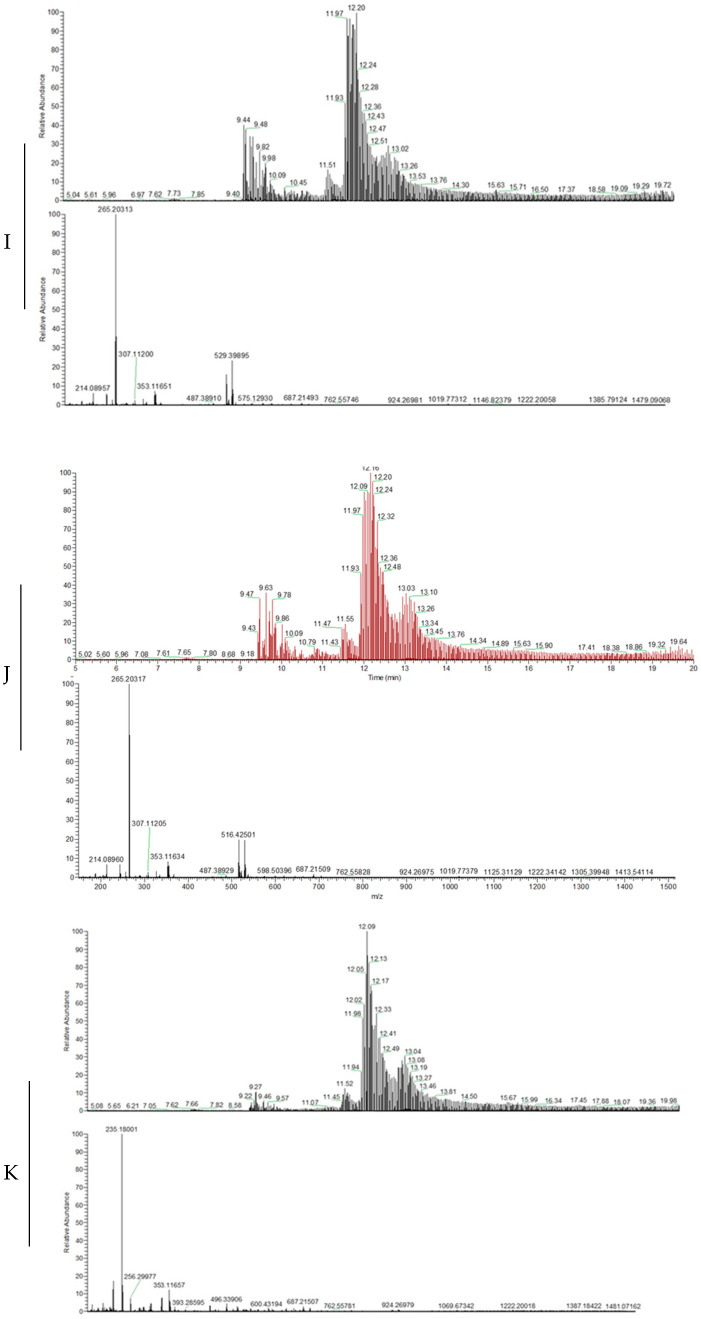

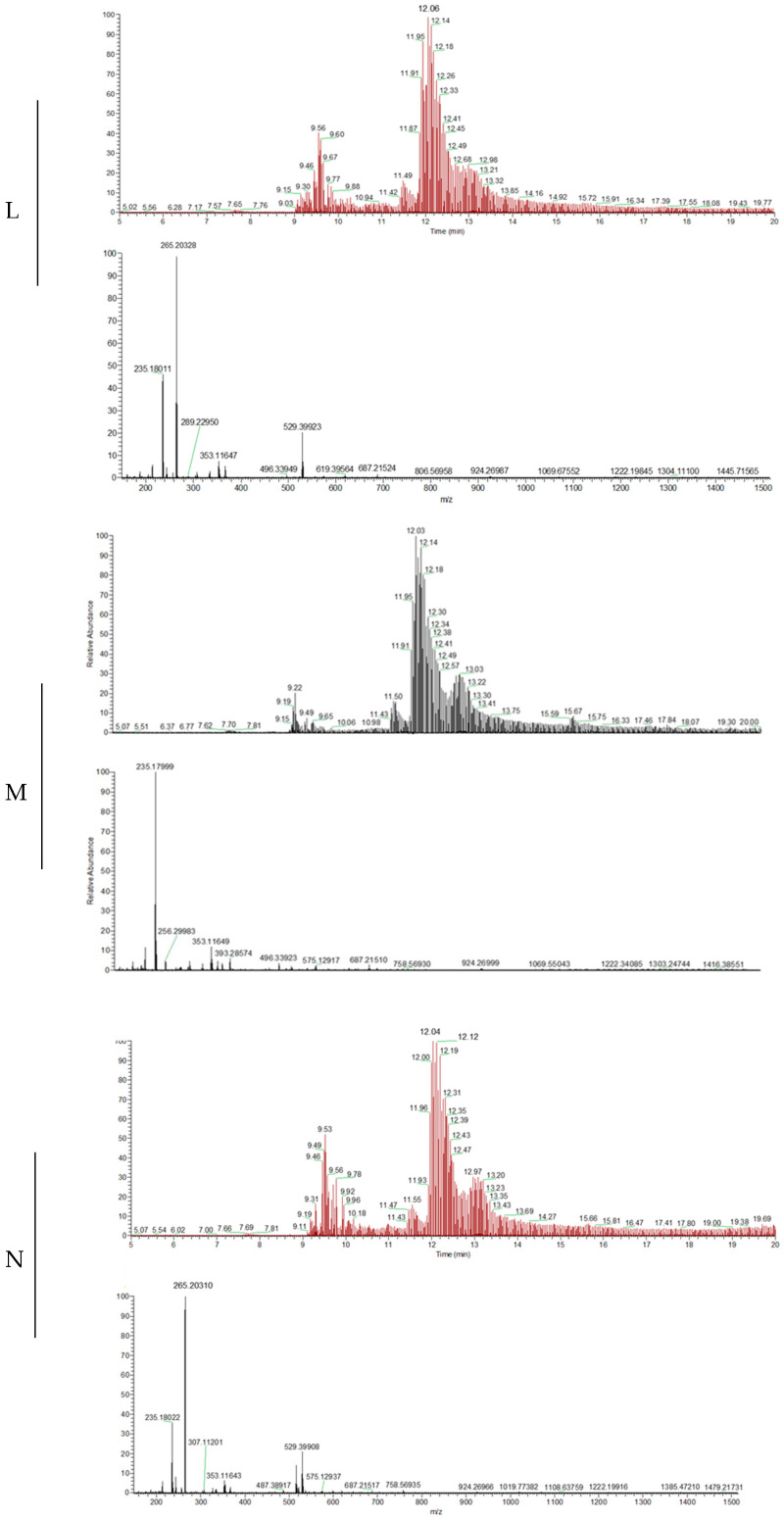

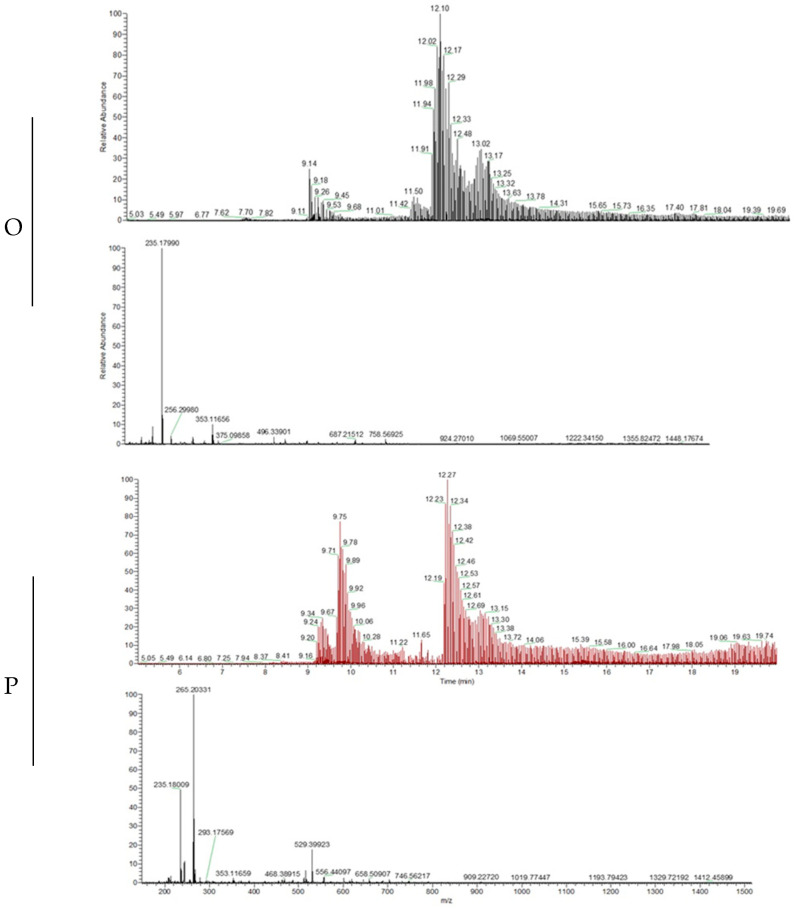
Representation of the LC-MS data for samples A to P, with the top image for each sample representing the DAD (UV–visible absorption spectra) and the bottom image showing the MS (ion fragmentation patterns). The DAD spectra reveal the UV–visible absorption profiles, while the MS spectra provide the characteristic ion fragmentation patterns, enabling the identification and quantification of the compounds present in each sample. The samples are (**A**): Control (DMEM) 24 h; (**B**): LIDO 500 µM 24 h; (**C**): LIDO 500 µM 48 h; (**D**): LIDO 500 µM 72 h; (**E**): PROP 500 µM 24 h; (**F**): PROP 500 µM 48 h; (**G**): PROP 500 µM 72 h; (**H**): ROC 500 µM 24 h; (**I**): ROC 500 µM 48 h; (**J**): ROC 500 µM 72 h; (**K**): LIDO 500 µM + PROP 500 µM 24 h; (**L**): LIDO 500 µM + ROC 500 µM 24 h; (**M**): LIDO 500 µM + PROP 500 µM 48 h; (**N**): LIDO 500 µM + ROC 500 µM 48 h; (**O**): LIDO 500 µM + PROP 500 µM 72 h; and (**P**): LIDO 500 µM + ROC 500 µM 72 h.

LC-MS was made to examine the supernatant of the samples whose combinations had the highest concentrations (Figure 14).

The LC-MS analysis successfully identified distinct chromatographic and mass spectrometric peaks corresponding to the different drugs. Lidocaine exhibited characteristic peaks around 9 min, likely indicating the presence of the drug or possible metabolites. Propofol showed only faint peaks, which may indicate its consumption by the cells. Rocuronium showed specific peaks between 9.5 and 12 min, marking its distinct metabolic signature.

### 2.3. Experimental Results: PBPK Model Simulations

In vitro studies using Hep G2 cells paved the way for subsequent in silico research, particularly in assessing the efficacy of combining lidocaine with propofol, paracetamol, and rocuronium. The in vitro approach allows a more detailed examination of the metabolic interactions and effects on CYP enzymes since these liver cells reproduce the metabolism of the human liver. Consequently, this method provides more accurate information on the pharmacokinetic behavior of drug combinations and the behavior of the drugs on the body. These computer models offer an overview and integrate and analyze data from multiple sources, providing a comprehensive view of the pharmacokinetic and pharmacodynamic properties of drugs and generating comprehensive predictions of a drug’s systemic performance, encompassing factors such as bioavailability, tissue perfusion, and elimination. The optimization process in this study involved maximizing bioavailability and minimizing potential drug–drug interactions (DDIs). Since this integrated modeling approach simulates interactions between various organs and tissues, providing a holistic view of the drug behavior in vivo, the objective was to identify the most efficient dosing combinations while adhering to constraints such as therapeutic dose ranges and metabolic factors. The decision variables involved were the drug concentrations and combinations, along with their respective pharmacokinetic parameters. This allowed for the simulation of potential interactions between lidocaine and the other three drugs.

Regarding the PK DDIs of lidocaine, little is known. Using the ADMET Predictor^®^ (Optimized Values), the physicochemical characteristics of lidocaine were determined and compared with the literature to examine, for the first time, the pharmacokinetic interactions between lidocaine and the other three drugs used with it in clinical practice (Table 2). 

To fill in the GastroPlus models for the other drugs, propofol, paracetamol, and rocuronium, the same methodology used for lidocaine was employed using ADMET Predictor^®^ (Optimized Values), the physicochemical characteristics of these drugs were determined and compared with the literature (Table 3).

#### 2.3.1. Metabolic Profile of Lidocaine, Propofol, Rocuronium, and Paracetamol

The metabolic profile of the four drugs was analyzed using ADMET Predictor^®^ (Table 4). The values presented in Table 4 represent the probability that lidocaine, propofol, paracetamol, and rocuronium act as substrates for specific enzymes in the *CYP450* system. These data were obtained based on predictions from the ADMET Predictor^®^ software, which estimates the potential affinity of each drug for certain *CYP450* isoenzymes. Thus, the values reflect the probable participation of each enzyme in drug metabolism. Among the nine enzymes of the CYP superfamily included in this computer program, lidocaine is a substrate of *CYP1A2*, *CYP2A6*, *CYP2B6*, *CYP2C8*, *CYP2C19*, *CYP2D6*, and *CYP3A4* with a probability of 91%, 57%, 57%, 59%, 81%, 87%, AND 66%, respectively. This prediction also suggests that this anesthetic is, with a probability of 31%, a *CYP2D6* inhibitor. As for propofol, it is a substrate of *CYP1A2*, *CYP2A6*, *CYP2B6*, *CYP2C8*, *CYP2C9*, *CYP2C19*, and *CYP2E1* with a probability of 79%, 84%, 88%, 56%, 50%, 66%, and 97%, respectively. This prediction also suggests that this anesthetic is, with a probability of 99%, a *CYP2B6* inhibitor. On the other hand, rocuronium is a substrate of *CYP3A4* with a probability of 67%. Finally, paracetamol is a substrate of *CYP1A2*, *CYP2A6*, and *CYP2E1*, with a probability of 66%, 45%, and 71%, respectively. This prediction also suggests that paracetamol is, with a probability of 68%, a *CYP2E1* inhibitor.

In addition to the sites of metabolization, data on the affinity of the enzyme for the substrate (Km, Michaelis–Menten constant), maximum rate of metabolization (Vmax), and intrinsic clearance (CL) are also presented.

#### 2.3.2. PBPK Model for Lidocaine

**Figure 15:**
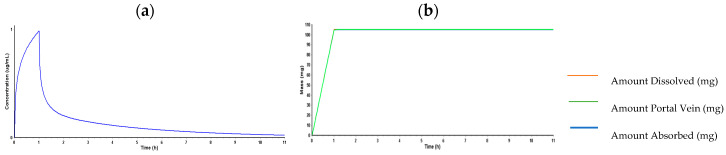
Pharmacokinetics of 105 mg lidocaine over a 24-h simulation in a 30-year-old American male: (**a**) an evaluation of the lidocaine plasma concentration over time, and (**b**) the amount of the drug in the portal vein, absorbed, and dissolved over time.

ADMET Predictor^®^ was used to estimate the pharmacokinetic parameters transferred to GastroPlus (Table 5) for lidocaine. Due to ADMET Predictor^®^’s restrictions, some variables (FDp, F, and hepatic Cmax) are not defined. According to the experimental section, the “Pharmacokinetics” function determined the PK parameters in a 30-year-old American male in good health who received 105 mg of lidocaine by IV (infusion). The PBPK model’s systemic distribution of lidocaine, as specified during a 24-h simulation, is depicted in Figure 15.

#### 2.3.3. PBPK Model for Propofol

**Figure 16:**
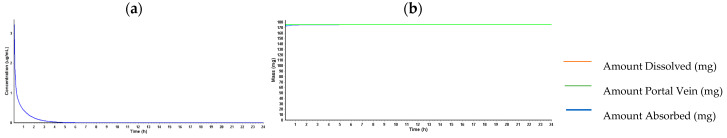
Pharmacokinetics of 175 mg propofol over a 24-h simulation in a 30-year-old American male: (**a**) an evaluation of the lidocaine plasma concentration over time, and (**b**) the amount of the drug in the portal vein, absorbed, and dissolved over time.

Next, ADMET Predictor^®^ was also used to estimate the pharmacokinetic parameters transferred to GastroPlus^®^ (Table 6) for propofol. Due to ADMET Predictor^®^’s restrictions, some variables (FDp, F, and hepatic Cmax) are not defined. According to the experimental section, the “Pharmacokinetics” function determined the PK parameters in a 30-year-old American male in good health who received 175 mg of propofol by IV. The PBPK model’s systemic distribution of propofol, as specified during a 24-h simulation, is depicted in Figure 16.

The PBPK model for propofol was developed, and it shows a rapid absorption, distribution, and elimination of the drug after intravenous (IV) administration. Propofol quickly reaches its Cmax, with a very short Tmax and efficient elimination, reflected by the AUC.

#### 2.3.4. PBPK Model for Paracetamol

**Figure 17:**
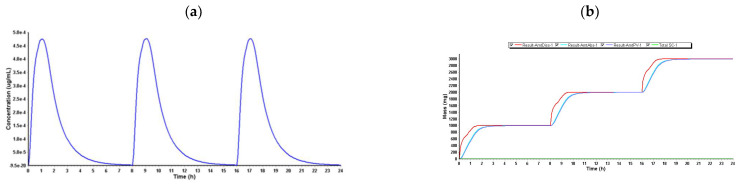
Pharmacokinetics of 1000 mg paracetamol q8h over a 24-h simulation in a 30-year-old American male: (**a**) an evaluation of the lidocaine plasma concentration over time, and (**b**) the amount of the drug in the portal vein, absorbed, and dissolved over time.

ADMET Predictor^®^ was used to estimate the pharmacokinetic parameters transferred to GastroPlus^®^ (Table 7) for paracetamol. Due to ADMET Predictor^®^’s restrictions, some variables (FDp, F, and hepatic Cmax) are not defined. According to the experimental section, the “Pharmacokinetics” function determined the PK parameters in a 30-year-old American male in good health who received 1000 mg of paracetamol tablets every 8 h (q8h). The PBPK model’s systemic distribution of paracetamol, as specified during a 24-h simulation, is depicted in Figure 17.

The PBPK model for paracetamol showed that, with standard doses of 1000 mg administered every 8 h, exposure to the drug remained constant, with no evidence of significant accumulation, as long as liver function was normal.

#### 2.3.5. PBPK Model for Rocuronium

**Figure 18:**
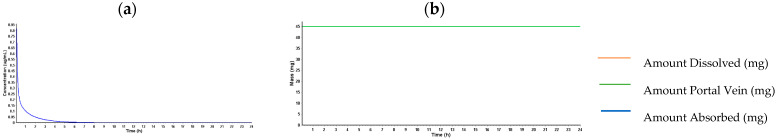
Pharmacokinetics of 45 mg rocuronium over a 24-h simulation in a 30-year-old American male: (**a**) an evaluation of the lidocaine plasma concentration over time, and (**b**) the amount of the drug in the portal vein, absorbed, and dissolved over time.

Lastly, ADMET Predictor^®^ was used to estimate the pharmacokinetic parameters transferred to GastroPlus^®^ (Table 8) for rocuronium. Due to ADMET Predictor^®^’s restrictions, some variables (FDp, F, and hepatic Cmax) are not defined. According to the experimental section, the “Pharmacokinetics” function determined the PK parameters in a 30-year-old American male in good health who received 45 mg of rocuronium by IV. The PBPK model’s systemic distribution of rocuronium, as specified during a 24-h simulation, is depicted in Figure 18.

The Cmax was reached quickly, reflecting its rapid action as a neuromuscular blocker, and a small AUC indicated efficient elimination without significant accumulation.

#### 2.3.6. Effect of Different Doses of Propofol on Lidocaine Pharmacokinetics

**Figure 19:**
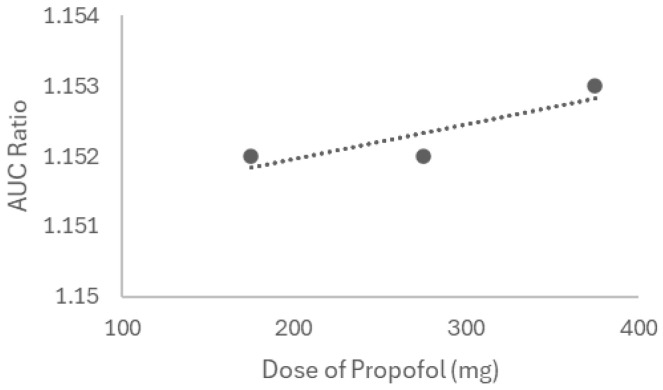
Effect of increasing the propofol dose on the AUC ratio of lidocaine, estimated by steady-state prediction in a 30-year-old healthy American male.

Aiming to examine the lidocaine–propofol interaction under different conditions, we first modeled three doses of propofol (175, 275, and 375 mg, IV) on lidocaine kinetics in a healthy 30-year-old American male. Both drugs were given once to simulate the real conditions. The interaction between lidocaine and propofol was first simulated in steady-state mode, with lidocaine as the victim and propofol as the perpetrator. Figure 19 reflects the interaction-derived AUC ratio as a function of the drug combination dosage, representing the relative change in AUC between conditions with and without drug interaction. For a propofol dose of 175 mg, an AUC ratio of 1.152 was recorded, indicating a weak interaction. When lidocaine is co-administered with 275 mg of propofol, the AUC stays at 1.152, and the highest dose (375 mg) corresponds to an AUC ratio of 1.153. Therefore, a proportional increase in the AUC ratio is observed as the dose of propofol increases.

Thereafter, the PK parameters of each combination (lidocaine 105 mg + propofol 175 mg, lidocaine 105 mg + propofol 275 mg, and lidocaine 105 mg + propofol 375 mg) were compared to the lidocaine baseline (administered alone) through dynamic simulation (Table 9). The AUC values reported in the table correspond to the calculated AUC for each specific drug combination and dose.

#### 2.3.7. Effect of Different Doses of Rocuronium on Lidocaine Pharmacokinetics

**Figure 20:**
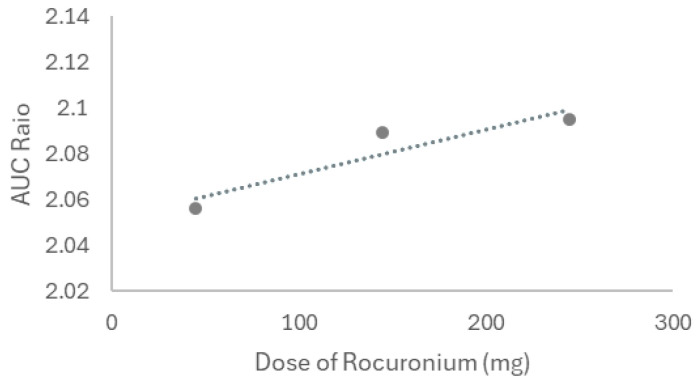
Effect of increasing the rocuronium dose on the AUC ratio of lidocaine estimated by steady-state prediction in a 30-year-old healthy American male.

Next, the same was performed for rocuronium, and to test the interaction under different conditions, we first modeled three doses of rocuronium (45, 145, and 245 mg, IV) on lidocaine kinetics in a healthy 30-year-old American male. Both drugs were given once to simulate the real conditions. The interaction between lidocaine and rocuronium was first simulated in steady-state mode, with lidocaine as the victim and rocuronium as the perpetrator. Figure 20 reflects the interaction-derived AUC ratio as a function of the drug combination dosage, representing the relative change in AUC between conditions with and without drug interaction. For a rocuronium dose of 45 mg, an AUC ratio of 2.056 was recorded, indicating a moderate interaction. When lidocaine is co-administered with 145 mg of rocuronium, the AUC goes to 2.089, and the highest dose (245 mg) corresponds to an AUC ratio of 2.095, all of them moderate and presenting moderate interactions.

Thereafter, the PK parameters of each combination (lidocaine 105 mg + rocuronium 45 mg, lidocaine 105 mg + rocuronium 145 mg, and lidocaine 105 mg + rocuronium 245 mg) were compared to the lidocaine baseline (administered alone) through dynamic simulation (Table 10). The AUC values reported in the table correspond to the calculated AUC for each specific drug combination and dose.

#### 2.3.8. Effect of Different Doses of Paracetamol on Lidocaine Pharmacokinetics

**Figure 21:**
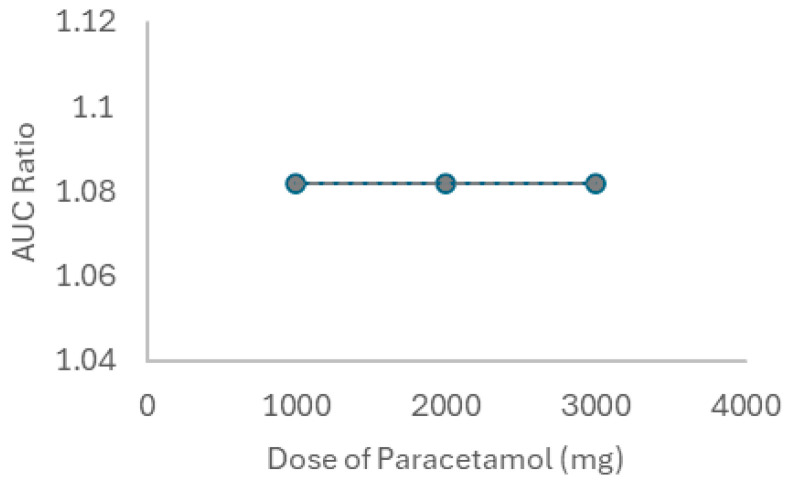
Effect of increasing the paracetamol dose on the AUC ratio of lidocaine estimated by steady-state prediction in a 30-year-old healthy American male.

To finalize this part, the same was performed for paracetamol, the third drug used in combination with lidocaine, and to test the interaction under different conditions, we first modeled three doses of paracetamol (1000, 2000, and 3000 mg, IV) on lidocaine kinetics in a healthy 30-year-old American male. Both drugs were given once to simulate the real conditions. The interaction between lidocaine and paracetamol was first simulated in the steady-state mode, with lidocaine as the victim and paracetamol as the perpetrator. Figure 21 reflects the interaction-derived AUC ratio as a function of the drug combination dosage, representing the relative change in AUC between conditions with and without drug interaction. For a rocuronium dose of 1000 mg, an AUC ratio of 1.082 was recorded, indicating no interaction. The value was registered for all paracetamol concentrations.

Thereafter, the PK parameters of each combination (lidocaine 105 mg + paracetamol 1000 mg, lidocaine 105 mg + paracetamol 2000 mg, and lidocaine 105 mg + paracetamol 3000 mg) were compared to the lidocaine baseline (administered alone) through dynamic simulation (Table 11). The AUC values reported in the table correspond to the calculated AUC for each specific drug combination and dose.

#### 2.3.9. Effect of Different Ages on Lidocaine Pharmacokinetics Co-Administered with Propofol

To investigate whether age had an influence on the co-administration of lidocaine and propofol, we simulated this therapeutic regimen in virtual male American subjects aged 10, 30, and 65 years (Table 12).

The results reveal that lidocaine concentrations are higher in 10-year-old children and 65-year-old elderly individuals. However, even though the AUC ratio is higher, the DDI classification remains the same for every different age, as a weak interaction.

#### 2.3.10. Effect of Different Ages on Lidocaine Pharmacokinetics Co-Administered with Rocuronium

To continue this investigation, the same process was performed. The co-administration of lidocaine and rocuronium was simulated in virtual male American subjects aged 10, 30, and 65 years (Table 13).

The results reveal that for every different age and dose, the interaction between lidocaine and rocuronium has the same classification, as moderate, and the AUC ratio is as big as the rocuronium dose.

#### 2.3.11. Effect of Different Ages on Lidocaine Pharmacokinetics Co-Administered with Paracetamol

To finalize this part of the investigation, the co-administration of lidocaine and paracetamol was tested, and we simulated this therapeutic regimen in virtual male American subjects aged 10, 30, and 65 years (Table 14).

The results reveal that lidocaine concentrations are reduced in 10-year-old children, and the AUC ratio remains equal for 30- and 65-year-old men. The DDI classification remains the same for every different age, with no interaction.

## 3. Discussion

This study was conducted to address the critical need for understanding drug–drug interactions (DDIs) between lidocaine and other commonly used perioperative medications, propofol, rocuronium, and paracetamol, given their widespread use and the potential for adverse interactions [57,58]. To achieve this, a combination of in vitro and in silico approaches were employed. Hep G2 cells were used to evaluate the cytotoxic effects of these drugs and their combinations, while PBPK modeling was utilized to predict the pharmacokinetic interactions in a systemic context.

The in vitro experiments conducted using the Hep G2 cell line provided insights into the cellular interactions between lidocaine, propofol, paracetamol, and rocuronium. The MTT assays (Figure 2, Figure 4, and Figure 6) revealed that after 24, 48, and 72 h of treatment, cell viability had varying impacts depending on the drug and concentration used. Typically, cell viability decreased as drug concentration increased [59,60].

After 24 h, treatment with lidocaine, propofol, paracetamol, and rocuronium at lower concentrations (50 and 100 µM) did not significantly affect the viability of the Hep G2 cells. However, by 48 h, the cytotoxic effects became more noticeable. Propofol, paracetamol, and rocuronium, especially at higher concentrations (250 µM and 500 µM), caused a significant decrease in cell viability, as shown by the increased statistical significance in the MTT assay results (Figure 4). These findings indicate that prolonged exposure to these drugs exacerbates their cytotoxic effects on hepatic cells, potentially due to accumulated metabolic stress or prolonged interaction with cellular pathways, and can also be fatal in certain situations of exposure [60,61,62,63]. Also, regarding paracetamol, at higher concentrations, such as 500 µM, cells might activate protective mechanisms in response to the drug exposure, such as better tolerance and resistance to the drugs [64,65], and this could explain the observed differences in cell viability. After 72 h, the results were similar to those at 48 h, with propofol and paracetamol continuing to show significant cytotoxic effects, particularly at higher concentrations (Figure 6). Interestingly, lidocaine and rocuronium, even at high doses, did not significantly affect cell viability over the 72 h, suggesting they have a lower level of toxicity. This could indicate a potential protective effect of these drugs under the tested conditions, saturation of receptors or enzymes, or even confounding factors that could be affecting the analyses since cell index is influenced by changes in multiple cell status, such as cell morphology, cell size, cell number, and adherence [63,66]. The microscopic analyses (Figure 3, Figure 5, and Figure 7) supported these viability findings by showing that, despite reductions in cell number, the overall morphology of the Hep G2 cells remained unchanged across different treatments and concentrations. This indicates that while certain drug combinations reduce cell viability, they do not necessarily induce morphological alterations, pointing to selective cytotoxicity rather than widespread cellular damage [67].

The combination studies (Figure 8, Figure 10, and Figure 12) revealed the interactive effects of these drugs. The pairing of lidocaine and propofol stood out, showing significant reductions in cell viability at 48 and 72 h, especially at higher concentrations (500 µM of each drug). This suggests a possible synergistic cytotoxic effect, which could have important implications for clinical settings where these drugs are co-administered [68]. Conversely, combinations of lidocaine with paracetamol and rocuronium did not produce such pronounced effects, implying that these combinations are less likely to result in adverse cellular interactions under the conditions tested. The observation that lidocaine alone does not significantly impact Hep G2 cell viability but does when used in combination with other drugs suggests that the negative effects on cellular health are likely due to the concomitant use, highlighting the potential for drug–drug interactions to exacerbate cytotoxicity [69,70]. So, these in vitro findings underscore the importance of considering drug concentrations and exposure times when assessing the safety of drug combinations in hepatic cells. The selective cytotoxicity observed with propofol and paracetamol, particularly in combination with lidocaine, highlights potential risks that may need to be addressed in clinical practices [71]. Future studies could explore the mechanistic underpinnings of these interactions, possibly involving oxidative stress pathways or CYP enzyme modulation, to further elucidate the observed effects.

The results from the LC-MS analysis are quite revealing. They show that both lidocaine and rocuronium have clear, recognizable patterns in their data, marked by distinct peaks. This tells us that these substances are present and can be tracked in the system effectively. However, the situation with propofol is different. It does not show a noticeable peak in the results. One possibility for this is that propofol might have been heavily taken up or used by the cells during the experiment. If the cells absorb a lot of propofol, it may be used up quickly or interacting with the cells in ways that we cannot directly see. This high uptake could explain why propofol seems to be more toxic to the cells. When the cells absorb a lot of propofol, it might disrupt their normal functions or put them under stress. This stress can lead to reduced cell viability, meaning the cells have a harder time surviving or functioning properly [72,73,74,75]. This aligns with the PBPK prediction of rapid tissue distribution and metabolism of propofol, which could explain its rapid cellular uptake observed in vitro and the corresponding decrease in cell viability.

So, while lidocaine and rocuronium show clear signs of their presence, the lack of a distinct peak for propofol suggests it is being heavily used or absorbed by the cells. This could be the reason behind its observed toxicity and lower cell viability, highlighting how propofol’s interaction with cells can affect their health.

Future studies could explore the mechanistic underpinnings of these interactions, possibly involving oxidative stress pathways or CYP enzyme modulation, to further elucidate the observed effects.

On the other hand, the PBPK model results developed to predict pharmacokinetic interactions between lidocaine, propofol, paracetamol, and rocuronium provide a comprehensive view of potential drug–drug interactions during the perioperative period [76,77]. The PBPK model developed for lidocaine using GastroPlus™ proved robust in predicting the pharmacokinetic properties of lidocaine, as shown in Table 5. Predicted values, such as time to maximum concentration (Tmax) and area under the curve (AUC), aligned well with the observed literature values, confirming the model’s accuracy. The simulation demonstrated a strong correlation between observed and predicted values, suggesting that the model is well-suited for predicting drug interactions involving lidocaine, and the accuracy of the PBPK predictions regarding lidocaine’s pharmacokinetics reinforces the relevance of the in vitro observations, confirming that the drug does not accumulate to toxic levels under typical clinical conditions. However, it is important to note that PBPK models undergo iterative optimization, where some parameters may require adjustments to better fit observed data. This can lead to variations from the literature values, especially when experimental conditions or population characteristics differ. The differences observed, particularly in Cmax values, are likely a result of these refinements, as PBPK models integrate multiple sources of data, including in vitro, in vivo, and in silico predictions. Occasionally, discrepancies may arise in some tables, such as Table 6, Table 7 and Table 8, because the literature data do not always align perfectly with the parameters used in the model. Our model is optimized for a specific dose and mode of administration, whereas the literature values are often generalized from broader pharmacological databases.

The analysis of interactions between propofol and lidocaine, described in Table 9 and visualized in Figure 19, revealed that the concomitant administration of propofol at increasing doses (175 mg, 275 mg, and 375 mg) resulted in a moderate increase in the AUC of lidocaine, indicating a weak interaction between the two drugs. This increase was more pronounced at higher doses of propofol, suggesting that propofol may partially inhibit the metabolism of lidocaine, thereby increasing its plasma concentration [78]. This pharmacokinetic interaction corresponds with the in vitro findings, where the combination of propofol and lidocaine resulted in enhanced cytotoxicity, suggesting that the interaction observed in silico may have functional implications at the cellular level. Also, DDIs involving propofol have been previously reported [79,80]. Table 10 and Figure 20 demonstrate that rocuronium, at different doses (45 mg, 145 mg, and 245 mg), exerts a moderate effect on the pharmacokinetics of lidocaine, with the AUC of lidocaine increasing as the dose of rocuronium increases. The AUC ratio ranged from 2.056 to 2.095, indicating a moderate interaction. This suggests that rocuronium may inhibit the metabolism of lidocaine, increasing its plasma concentration. This interaction could be critical in clinical contexts where both drugs are administered, potentially prolonging the anesthetic effect of lidocaine and increasing the risk of toxicity [81]. Also, it has been demonstrated that the clinical duration of rocuronium is prolonged when followed by maintenance doses of other neuromuscular blocking agents, which can produce a synergistic effect, enhancing and extending the blockade [82,83].

As for paracetamol and lidocaine, shown in Table 11 and Figure 21, there were no interactions, with the AUC ratio remaining around 1.082 regardless of the dose of paracetamol administered (1000 mg, 2000 mg, or 3000 mg). This indicates that paracetamol has a minimal effect on the pharmacokinetics of lidocaine, suggesting that these two drugs can be co-administered with a relatively low risk of significant interactions. This result is relevant for clinical practice as paracetamol is commonly used for pain management in combination with local anesthetics like lidocaine [84,85], and this lack of significant cytotoxicity is consistent with the in vitro results, which indicated no relevant toxicity to the cells between lidocaine and paracetamol, supporting their safe co-administration.

The influence of age on drug interactions between lidocaine and other drugs was explored in Table 12, Table 13 and Table 14. The simulation for different age groups (10, 30, and 65 years) revealed that while the effect of propofol on lidocaine was classified as a weak interaction across all ages, rocuronium showed a moderate interaction, particularly in older patients. In contrast, paracetamol showed no significant alteration in interactions with lidocaine, regardless of age. These findings are crucial for dose adjustment in pediatric and geriatric populations, where physiological changes can impact the pharmacokinetics and safety of drugs, since age is a crucial factor in hepatic clearance alterations, as the liver’s ability to metabolize and eliminate drugs depends on its efficiency in removing the drug from systemic circulation, drug uptake by hepatocytes, and enzyme activity, all of which change over time [85,86,87,88].

While lidocaine demonstrated significant interactions with propofol and rocuronium, paracetamol seems to be relatively safe for co-administration. The model also emphasized the importance of considering patient age when predicting interactions and adjusting treatment, especially in more vulnerable populations. These findings show that the PBPK model developed is a valuable tool for predicting drug–drug interactions and adjusting dosages in clinical settings. However, given the scale of certain parameter discrepancies, additional experimental validation, including in vivo pharmacokinetic studies, would further strengthen confidence in the model’s predictive accuracy. It is important to highlight that the quality and reliability of PBPK simulations hinge on the model itself and the data it is based on, as well as what the simulation aims to achieve. Simulations come with their own set of prediction errors and uncertainties, where some parameters may need to be adjusted to better fit observed data, potentially leading to differences from the literature values. These variations highlight the importance of careful interpretation and further validation with experimental data, so it is crucial to interpret their results thoughtfully and in the right context. PBPK models often rely on population averages from healthy adults, which can lead to inaccurate predictions due to individual variability and the complexities of comorbidities, making them less applicable to children, the elderly, or patients with chronic diseases. Additionally, findings from these simulations should be backed up by real experimental data and should not replace results from well-designed studies as the primary evidence [89,90]. On the other hand, due to the low levels of CYP enzymes in Hep G2 cells, the cytotoxicity of various compounds may have been overlooked or underestimated compared to the effects observed in primary human hepatocytes, highlighting a limitation of using this cell line for toxicological studies [56].

So, it is essential to combine both in vitro and in silico studies to fully assess the safety and effectiveness of using multiple drugs together, as each method offers unique and complementary insights. In vitro experiments, like those on the Hep G2 cells, provide direct observations of how cells respond to drug combinations, revealing potential toxic effects that may not be obvious from pharmacokinetic data alone. On the other hand, in silico modeling, such as PBPK simulations, predicts how drugs interact within the body at a systemic level, considering factors like metabolism, distribution, and enzyme inhibition. While in vitro studies highlight the cellular-level impacts, in silico models help anticipate broader pharmacokinetic interactions. By using both methods, we obtained a more complete understanding, which helps ensure safer and better-informed clinical decisions [91,92]. Allied to these studies, studies on real patients will provide valuable information that laboratory models cannot fully capture, although our results suggest starting with lower doses of propofol and rocuronium when used with lidocaine, especially in longer surgeries, to ensure safer anesthetic management adapted to different patient populations and adjusting as the surgery proceeds.

## 4. Materials and Methods

### 4.1. In Vitro Model

#### 4.1.1. Cell Line

Hep G2 cells are a cell line exhibiting epithelial-like morphology that was isolated from a hepatocellular carcinoma of a 15-year-old, white, male youth with liver cancer. As Hep G2 cells retain most of the metabolic functions of normal hepatocytes, this makes them a good model for studying the toxic effects of substances like heavy metals, nanoparticles, and drugs in vitro, as performed in this study. They also show greater similarity to the human liver compared to other cell lines when it comes to the expression of cellular proteins. Additionally, some studies have demonstrated that Hep G2 cells can mimic human hepatocytes in terms of *CYP1A2* and *CYP3A4* inducibility, suggesting that they can serve as a valuable alternative model for evaluating CYP induction [50,93,94]. The Hep G2 cells were cultured. Briefly, the cells were kept in DMEM medium supplemented with 10% FBS and 1% antibiotic solution (10,000 U/mL penicillin; 10,000 μg/mL streptomycin) in 25-cm^2^ flasks, under a humidified 5% CO_2_ atmosphere at 37 °C, and subjected to regular medium changes (every 2–3 days). Whenever the cells reached 80% confluence, they were sub-cultured by trypsinization. For the cytotoxicity assays, the cells were seeded in 96-well plates at a density of 50 × 10^3^ cells per well in a volume of 200 μL of complete medium and left to adhere overnight.

#### 4.1.2. Cell Culture

The Hep G2 cells (obtained from the American Type Culture Collection, Manassas, VA, USA) were cultured at 37 °C in a 95% air and 5% CO_2_ environment. The cell culture medium was created by mixing up 1% penicillin (1000 U/mL)/streptomycin (10 mg/mL) and 10% FBS, added to DMEM. Trypsin (0.25% trypsin-EDTA) was used to detach the cells before each experiment. The cells were then seeded at a density of 50 × 10^3^ cells/cm^2^ in 96-well plates for viability experiments after being centrifuged for five minutes at 1100 rpm for 5 min using a Hettich centrifuge (Tuttlingen, Germany).

#### 4.1.3. Cell Treatment

Lidocaine, propofol, paracetamol, and rocuronium were dissolved in DMEM, according to the concentrations defined, to test 50, 100, 250, and 500 μM of each drug in the cells. The controls were the cell culture medium. All the treatments were tested for a period of 24, 48, and 72 h after cell attachment to the plates. For all the combinations tested, both agents were added simultaneously.

#### 4.1.4. Cell Morphology Assessments

After the drug treatment, the Hep G2 cells’ morphological characteristics were assessed and documented using a Leica DMI 6000B Automated Microscope coupled with a Leica DFC350 FX camera (Leica Microsystems, Wetzlar, Germany). The plate containing the cells was placed under the microscope, and Leica Las X imaging software (v3.7.4) (LeicaMicrosystems, Wetzlar, Germany) was used to examine the cell images on a computer. The assessments were qualitative.

#### 4.1.5. Cell Viability Assays

Using MTT assays, cellular viability was evaluated at 24, 48, and 72 h following the start of the cell treatments. After the culture media was removed, 100 μL of MTT solution (0.5 mg/mL in PBS) was added to each well. After that, the cells were kept out of the light for 3 h. Following aspiration of the MTT solution, 100 μL of DMSO was added to each well. Ultimately, an automated microplate reader (Tecan Infinite M200, Zurich, Switzerland) was used to detect absorbance values at 570 nm.

#### 4.1.6. Statistical and Data Analyses

The results were presented as the mean ± SD (standard error of the mean) of three different cell culture preparations. Utilizing one-way ANOVA tests and Dunnett’s multiple comparisons test, statistical analyses were carried out between the treatment and control conditions. A *p*-value < 0.05 indicated that the differences were significant. GraphPad Prism 8 software was used to perform all statistical analyses and create graphs (San Diego, CA, USA).

#### 4.1.7. LC-MS Method Development

An amount of 5 uL of each sample was separated on an HPLC Vanquish (Thermo Fischer Scientific, Bremen, Germany) using an Accucore RP-MS column (Thermo Fischer Scientific, Vilnius, Lithuania) with a 2.6 µm particle size and the dimensions 100 mm × 2.1 mm. The samples were eluted over a gradient of 100% solvent A (H20 with 0.1% *v*/*v* HCOOH) and 100% Solvent B (ACN with 0.1% *v*/*v* HCOOH) for 15 min at a flow rate of 0.150 mL/min. The column temperature was 25 °C. The analyses were performed on an Orbitrap Exploris 120 mass spectrometer (Thermo Fischer Scientific, Bremen, Germany) controlled by Orbitrap Exploris Tune Application 2.0.185.35 and Xcalibur 4.4.16.14. The capillary voltage of the electrospray ionization source (ESI) was set to 3.5 kV and 2.4 kV for positive and negative modes. The capillary temperature was 300 °C. The sheath gas and auxiliary gas flow rates were at 50 and 10 (arbitrary units as provided by the software settings). The resolution of the MS scan was 60,000. Data-dependent MS/MS was performed on HCD using nitrogen as a gas with collision energy settings of 35 V. The *m*/*z* range was 100–800 Da. The resolution of the SIM MS scan was 60,000, with center masses at *m*/*z* 179.143, 235.1805, 529.4. MS data handling software (Xcalibur QualBrowser software (version 3.1), Thermo Fischer Scientific) was used to search for predicted metabolites by their *m*/*z* value and MS/MS value.

### 4.2. PBPK Model

#### 4.2.1. Prediction of Pharmacokinetic and Physicochemical Properties of Lidocaine, Paracetamol, Propofol, and Rocuronium

Using ADMET Predictor^®^ (Version 10.4; Simulation Plus Inc., Lancaster, CA, USA), a software program that precisely predicts several aspects of compounds, including physicochemical and pharmacokinetic properties (PK properties), all four drugs were described by their respective physicochemical and PK properties. Comprising three modules (compound, physiology, and pharmacokinetics), this application computes the fraction of the drug dose absorbed in each intestinal compartment using the ACAT model. The drug’s chemical structures were created using MedChem Designer (Version 5.5; Simulation Plus Inc., Lancaster, CA, USA), and the MOL file format was subsequently imported into ADMET Predictor^®^. This software program was used to evaluate parameters, including Log P, molecular weight, solubility, CYP-mediated metabolism and transport, human jejunum effective permeability (Peff), diffusion coefficient (Diff. Coeff.), and blood–brain barrier (BBB) permeability, and these parameters were then used as input for GastroPlus. The PKs of the drugs were simulated using an ADMET Predictor^®^ functionality (%Fa and %Fb calculator) with various IV dosages administered over 24 h. The outputs included key PK parameters, such as the fraction absorbed (Fa), the fraction available in the portal vein (FDp), and bioavailability (F). It also estimated the Tmax (time to peak concentration), the Cmax (maximum plasma concentration), the AUC (area under the curve), and the contributions of hepatic metabolism by CYP enzymes. These values were then input into GastroPlus to build the PBPK model.

#### 4.2.2. Potential Drug Interactions with Lidocaine Based on CYP

**Figure 22:**
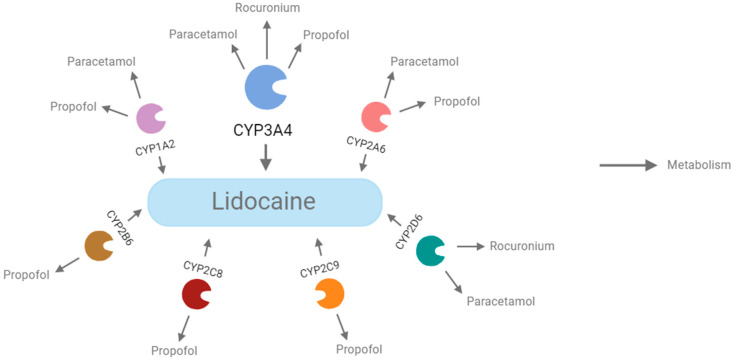
Network of the drug–drug interactions that were incorporated in the model, based on Table 1, created in BioRender on 26 December 2024.

In addition to the literature review of the CYPs of the drugs registered in Table 1, the MOL file for each drug obtained with MedChem Designer was also uploaded to ADMET Predictor^®^. The CYPs obtained from each of the searches from the literature and predicted by ADMET Predictor^®^ were mostly corresponding (Figure 22).

#### 4.2.3. Development of a PBPK Model for Lidocaine

The GastroPlus software (Version 9.8.3; Simulation Plus Inc., Lancaster, CA, USA) was used to create the PKBK model for lidocaine. All of the information used in this software, including the drug’s chemical structure and its physicochemical and PK parameters, was previously computed using ADMET Predictor^®^. Therefore, all other program sections used the projected values, except the ‘Gut Physiology’ tab, which specifies individual features. An IV of 105 mg of lidocaine was used to simulate its properties. ADMET Predictor^®^ was used to obtain the observed bioavailability values (Fa, fraction absorbed; FDp, fraction of drug concentration in the portal vein; and F, fraction of drug concentration in the blood), Tmax (time to peak drug concentration), AUC (area under the curve), CmaxLiver (maximum plasma concentration measured in the liver), and the time required for maximum plasma concentration. The parameters of the compartmental PK model of a 30-year-old healthy American virtual patient were established based on the disposition of the drug. The 24-h simulation produced quantitative and graphical results regarding the properties of PKs. Among the key outputs were plasma concentration–time profiles, tissue distribution, clearance rates, and drug exposure predictions. Following this confirmation, several aspects of the subjects, such as age, weight, and state of health, as well as drug doses, were modeled for the DDI simulations, which were designed to be simulated by DDIs. The body mass index (BMI) scale was used to determine weight: a BMI of 18.5–24.9 is considered normal, a BMI of 25–29.9 is overweight, and a BMI of 30 or higher is obese. Also, different ages were simulated.

#### 4.2.4. PBPK Model Validation

To confirm the accuracy of the model, data from the literature were compared with the PK parameter values that were derived from the established models [95,96]. To further ensure confidence in the PBPKs, a visual examination of the plots of the plasma concentration profile was carried out. Thus, the PBPK models were verified.

#### 4.2.5. DDI Simulations

DDIs for lidocaine and the other three drugs were carried out in the GastroPlus DDI module utilizing the steady-state mode and dynamic simulation. The inputs for these simulations included the individual PBPK models developed in GastroPlus for each drug. Additionally, parameters, such as drug dosing regimens, administration routes, and enzyme kinetics, were incorporated to accurately model the interactions. Since lidocaine is a drug that is combined with all other drugs, it is the victim, and the other drugs are the perpetrators, as the role of the victim or perpetrator depends on which drug is most affected by changes in its metabolism due to the presence of other drugs. In the case of lidocaine, as it is a drug that is always administered in combination with others, it becomes the most susceptible to changes in its metabolism and therefore is considered the victim in drug interactions, as its metabolism is influenced by the actions of the other three drugs. Based on this assumption, the previously computed dataset was used as input for the DDI predictions. We conducted the simulations using the previously created PBPK models. First, using the PBPK model of a healthy 30-year-old man, the interaction between propofol and lidocaine was predicted for 24 h in both dynamic and steady-state modes. The same process was then replicated with paracetamol and rocuronium. Subsequently, we used the other PBPK models (different ages) to investigate the DDIs under different conditions. These predictions were conducted in steady-state mode. The outputs of these simulations included the AUC (area under the curve) ratio of lidocaine with and without each interacting drug, changes in Cmax (maximum plasma concentration), and PK parameters compared between drugs alone and in combination.

The classification of DDIs is based on the AUC ratio in the presence or absence of the perpetrator and is categorized as no interaction, weak, moderate, or strong. The interaction is weak, with an AUC ratio ranging from 1.25 to 2. An AUC ratio in the range of 2 to 5 indicates a moderate interaction. AUC ratios above 5 indicate a strong interaction.

## 5. Conclusions

This work used both in-vitro studies of drug–drug interactions (DDIs) as well physiologically based pharmacokinetics (PBPK modeling) to predict and evaluate DDIs between lidocaine with three frequently co-administered drugs in perioperative care: propofol, paracetamol, or rocuronium. In vitro data from Hep G2 cells can be incorporated as well with PBPK models to evaluate the pharmacokinetic and cytotoxic interactions between these drugs. The findings suggest that clinicians can safely use these drug combinations in most clinical scenarios, but they should remain vigilant for potential interactions, particularly with propofol and rocuronium at higher doses. As a predictive tool, PBPK modeling was useful in providing a better knowledge of drug interactions and could help develop safer dosing regimens.

This work marks an important step in understanding pharmacokinetic interactions in a clinical setting. The PBPK tools used in this study can help optimize drug concentration levels by validating and refining models with real-world patient data. This is crucial to ensure the safe and effective use of these drug combinations in practice and to guide future clinical studies.

However, to build on these findings, it will be essential to conduct clinical trials with real patients, especially in surgical settings. This would allow us to directly observe how these drug interactions affect patients during and after surgery, providing practical insights that go beyond simulations. Future research should also explore these interactions in different patient populations, such as children, the elderly, and individuals with impaired liver or kidney function. Understanding how metabolic differences impact these interactions will help personalize treatment and ensure safer and more effective drug administration for a variety of patients.

This study also suggests some recommendations for future research and clinical practice. First, start with lower doses of propofol and rocuronium when used with lidocaine, especially for longer surgeries, and adjust based on how patients respond. It is important to regularly check liver function and enzyme levels to avoid toxicity, particularly in at-risk patients. Clinical trials should include diverse groups, like children and the elderly, to create tailored treatment plans. Lastly, conducting pilot studies during surgeries will help validate these findings and refine dosing strategies for safer anesthesia management.

Finally, this study provides important information in understanding the pharmacokinetic interactions of lidocaine with commonly used perioperative agents overall. In conclusion, with all of this knowledge, we may be able to improve patient care by personalizing it and optimizing anesthetic procedures.

## Figures and Tables

**Table 1:** A summary of the cytochrome P450 (CYP) interactions for lidocaine, propofol, paracetamol, and rocuronium, indicating whether each drug acts as an inhibitor, substrate, or inducer [39,40,41,42,43,44].

Drug	*CYP1A2*	*CYP2A6*	*CYP2B6*	*CYP2C8*	*CYP2C9*	*CYP2D6*	*CYP3A4*
Lidocaine	x (I,S)	x (S)	x (S)	x (S)	x (S)	x (S,I)	x (S)
Paracetamol	x (S)					x (S)	x (ID)
Propofol	x (I)		x (S)	x (S)	x (S)		x (S)
Rocuronium						x (S)	x (S)

I: Inhibitor; S: Substrate; ID: Inducer.

**Table 2:** Predicted and optimized physiochemical properties of lidocaine.

Physiochemical Properties	Literature Value	Optimized Value	Reference
Log P	1.81	2.288	[39,40,41,42,43,44]
Ionization Constant	7.86	7.84	
Molecular Weight (g/mol)	234.337	234.344	
Water Solubility (mg/mL)	9	1.793	
Diff. Coeff. (cm^2^/s·10^5^)	ND	0.782	ND
Peff (cm/s·10^4^)	0.9	3.754	[39,40,41,42,43,44]
BBB Penetration	ND	High (99%)	ND

Diff. Coeff, differential coefficient; Peff, effective human jejunal permeability; BBB, blood–brain barrier; ND, not defined.

**Table 3:** Predicted and optimized physiochemical properties of propofol, paracetamol, and rocuronium.

	Propofol		Paracetamol		Rocuronium		
Physiochemical Properties	Literature Value	Optimized Value	Literature Value	Optimized Value	Literature Value	Optimized Value	Reference
Log P	3.79	3.386	0.51	0.449	2.71	0.589	[39,40,41,42,43,44]
Ionization Constant	11.67	11.07	9.46	12.17	14.59	6.74	
Molecular Weight (g/mol)	178.271	178.271	151.163	151.163	529.774	529.774	
Water Solubility (mg/mL)	4.4	0.150	4.15	9.448	2.84 × 10^5^	0.056	
Diff. Coeff. (cm^2^/s·10^5^)	ND	0.901	ND	1.114	ND	0.509	
Peff (cm/s·10^4^)	1	9.1	0.9	3.778	1	0.402	
BBB Penetration	ND	High (99%)	ND	High (99%)	ND	Low (53%)	

Diff. Coeff, differential coefficient; Peff, effective human jejunal permeability; BBB, blood–brain barrier; ND, not defined.

**Table 4:** Metabolic profile of lidocaine, propofol, paracetamol, and rocuronium, predicted using ADMET Predictor^®^.

Drug	CYP Enzime	Inhibitor	Substrate	Km	V_max_	CL
Lidocaine	1A2	ND	Yes (91%)	382.404	40.878	5.559
	2A6	ND	Yes (57%)	ND	ND	ND
	2B6	ND	Yes (57%)	ND	ND	ND
	2C8	ND	Yes (59%)	ND	ND	ND
	2C9	No (97%)	No (86%)	NS	NS	NS
	2C19	No (96%)	Yes (81%)	79.951	147.163	25.769
	2D6	Yes (31%)	Yes (87%)	51.519	12.605	1.957
	2E1	No (96%)	No (98%)	ND	ND	ND
	3A4	No (96%)	Yes (66%)	288.766	16.695	6.417
Propofol	1A2	No (48%)	Yes (79%)	15.993	10.968	35.662
	2A6	No (99%)	Yes (84%)	ND	ND	ND
	2B6	Yes (99%)	Yes (88%)	ND	ND	ND
	2C8	No (82%)	Yes (56%)	ND	ND	ND
	2C9	No (62%)	Yes (50%)	228.963	117.582	37.488
	2C19	No (52%)	Yes (66%)	190.718	57.724	4.237
	2D6	No (74%)	No (76%)	NS	NS	NS
	2E1	No (69%)	Yes (97%)	ND	ND	ND
	3A4	No (96%)	No (56%)	NS	NS	NS
Rocuronium	1A2	No (96%)	No (97%)	NS	NS	NS
	2A6	No (99%)	No (98%)	ND	ND	ND
	2B6	No (72%)	No (75%)	ND	ND	ND
	2C8	No (65%)	No (94%)	ND	ND	ND
	2C9	No (97%)	No (99%)	NS	NS	NS
	2C19	No (96%)	No (95%)	NS	NS	NS
	2D6	No (90%)	No (60%)	NS	NS	NS
	2E1	No (99%)	No (93%)	ND	ND	ND
	3A4	No (85%)	Yes (67%)	21.157	73.479	385.502
Paracetamol	1A2	No (96%)	Yes (66%)	1465.930	46.959	1.666
	2A6	No (99%)	Yes (45%)	ND	ND	ND
	2B6	No (86%)	No (92%)	ND	ND	ND
	2C8	No (97%)	No (81%)	ND	ND	ND
	2C9	No (97%)	No (87%)	NS	NS	NS
	2C19	No (96%)	No (66%)	NS	NS	NS
	2D6	No (97%)	No (69%)	NS	NS	NS
	2E1	Yes (68%)	Yes (71%)	ND	ND	ND
	3A4	No (96%)	No (83%)	NS	NS	NS

ND: Not Defined; NS: No Substrate.

**Table 5:** Observed (ADMET Predictor^®^) and estimated (GastroPlus) pharmacokinetic properties of 105 mg lidocaine administered after a 24-h simulation.

Pharmacokinetic Parameters	Observed Values	Estimated Value
Fa (%)	100	99.983
FDp (%)	ND	99.573
F (%)	ND	99.883
Cmax (µg/mL)	0.728	0.98816
Tmax (h)	1	1
AUC0-inf (ng·h/mL)	1926	1884.4
AUC0-t (ng·h/mL)	1926	1785.1
Cmax liver (µg/mL)	ND	1.0774

ND: Not defined.

**Table 6:** Predicted (ADMET Predictor^®^) and estimated (GastroPlus^®^) pharmacokinetic properties of 175 mg propofol administered to a 30-year-old man after a 24-h simulation.

Pharmacokinetic Parameters	Predicted Values	Estimated Value
Fa (%)	100	99.994
FDp (%)	ND	99.994
F (%)	ND	100
Cmax (µg/mL)	0.29861	41.482
Tmax (h)	0.2	0.2
AUC0-inf (ng·h/mL)	4171.8	2074.5
AUC0-t (ng·h/mL)	4171.8	2074.5
Cmax liver (µg/mL)	ND	7.14 × 10^−6^

ND: Not defined.

**Table 7:** Predicted (ADMET Predictor^®^) and estimated (GastroPlus^®^) pharmacokinetic properties of 1000 mg paracetamol q8h administered to a 30-year-old man after a 24-h simulation.

Pharmacokinetic Parameters	Predicted Values	Estimated Value
Fa (%)	99.9	99.892
FDp (%)	ND	99.888
F (%)	ND	ND
Cmax (μg/mL)	8.5445	4.764 × 10^−4^
Tmax (h)	0.91	17.04
AUC0-inf (ng·h/mL)	16.466	2.97 × 10^−3^
AUC0-t (ng·h/mL)	16.466	2.968 × 10^−3^
Cmax liver (µg/mL)	ND	7.558 × 10^−4^

ND: Not defined.

**Table 8:** Predicted (ADMET Predictor^®^) and estimated (GastroPlus^®^) pharmacokinetic properties of 45 mg rocuronium administered to a 30-year-old man after a 24-h simulation.

Pharmacokinetic Parameters	Predicted Values	Estimated Value
Fa (%)	100	99.946
FDp (%)	ND	99.946
F (%)	ND	100
Cmax (μg/mL)	0.04103	10.667
Tmax (h)	0	0
AUC0-inf (ng·h/mL)	551.31	533.44
AUC0-t (ng·h/mL)	551.31	533.44
Cmax liver (µg/mL)	ND	1.005 × 10^−6^

**Table 9:** Effect of increasing the propofol dose on the pharmacokinetics of lidocaine. The pharmacokinetic parameters were estimated by dynamic simulation for 24 h in a 30-year-old healthy American male.

Compound	Fa (%)	FDp (%)	F (%)	Cmax (µg/mL)	Tmax (h)	AUC0-t (ng·h/mL)	AUC0-Inf (ng·h/mL)
Lidocaine baseline 105 mg	99.983	99.573	99.883	0.9816	1	1884.4	1785.1
Lidocaine 105 mg + Propofol 175 mg	99.99	99.59	99.88	43.85	0	1962.6	1966.6
Lidocaine 105 mg + Propofol 275 mg	99.99	99.59	99.88	43.85	0	1965.3	1969.4
Lidocaine 105 mg + Propofol 375 mg	99.99	99.59	99.87	43.85	0	1968	1972

**Table 10:** Effect of increasing the rocuronium dose on the pharmacokinetics of lidocaine. The pharmacokinetic parameters were estimated by dynamic simulation for 24 h in a 30-year-old healthy American male.

Compound	Fa (%)	FDp (%)	F (%)	Cmax (µg/mL)	Tmax (h)	AUC0-t (ng·h/mL)	AUC0-Inf (ng·h/mL)
Lidocaine baseline 105 mg	99.983	99.573	99.883	0.9816	1	1884.4	1785.1
Lidocaine 105 mg + Rocuronium 45 mg	99.96	99.96	100	18.96	0	948.3	948.3
Lidocaine 105 mg + Rocuronium 145 mg	99.96	99.96	100	18.96	0	948.3	948.3
Lidocaine 105 mg + Rocuronium 245 mg	99.96	99.96	100	18.96	0	948.3	948.3

**Table 11:** Effect of increasing the paracetamol dose on the pharmacokinetics of lidocaine. The pharmacokinetics parameters were estimated by dynamic simulation for 24 h in a 30-year-old healthy American male.

Compound	Fa (%)	FDp (%)	F (%)	Cmax (µg/mL)	Tmax (h)	AUC0-t (ng·h/mL)	AUC0-Inf (ng·h/mL)
Lidocaine baseline 105 mg	99.983	99.573	99.883	0.9816	1	1884.4	1785.1
Lidocaine 105 mg + Paracetamol 1000 mg	99.96	99.96	99.99	24.89	0	1244.6	1244.6
Lidocaine 105 mg + Paracetamol 2000 mg	99.96	99.96	99.99	24.89	0	1244.6	1244.6
Lidocaine 105 mg + Paracetamol 3000 mg	99.96	99.96	99.99	24.89	0	1244.6	1244.6

**Table 12:** Interaction of the different doses (175, 275, and 375 mg) of propofol on the pharmacokinetics of lidocaine in 10-, 30-, and 65-year-old virtual subjects.

Dosing Regimen	AUC Ratio			DDI Classification		
Age	10	30	65	10	30	65
Lidocaine with Propofol 175 mg	1.567	1.152	1.723	W	W	W
Lidocaine with Propofol 275 mg	1.567	1.152	1.724	W	W	W
Lidocaine with Propofol 375 mg	1.567	1.153	1.724	W	W	W

W: weak.

**Table 13:** Interaction of the different doses (45, 145, and 245 mg) of rocuronium on the pharmacokinetics of lidocaine in 10-, 30-, and 65-year-old virtual subjects.

Dosing Regimen	AUC Ratio			DDI Classification		
Age	10	30	65	10	30	65
Lidocaine with Rocuronium 45 mg	2.019	2.056	2.024	M	M	M
Lidocaine with Rocuronium 145 mg	2.077	2.089	2.054	M	M	M
Lidocaine with Rocuronium 245 mg	2.088	2.095	2.074	M	M	M

M: moderate.

**Table 14:** Interaction of the different doses (1000, 2000, and 3000 mg) of paracetamol on the pharmacokinetics of lidocaine in 10-, 30-, and 65-year-old virtual subjects.

Dosing Regimen	AUC Ratio			DDI Classification		
Age	10	30	65	10	30	65
Lidocaine with Paracetamol 1000 mg	1.071	1.082	1.082	N/I	N/I	N/I
Lidocaine with Paracetamol 2000 mg	1.071	1.082	1.082	N/I	N/I	N/I
Lidocaine with Paracetamol 3000 mg	1.071	1.082	1.082	N/I	N/I	N/I

N/I: no interaction.

## Data Availability

No new data were created or analyzed in this study. Data sharing is not applicable to this article.
